# Multiregion profiling of genomic and transcriptional heterogeneity in head and neck squamous-cell carcinoma

**DOI:** 10.1016/j.esmoop.2026.108367

**Published:** 2026-09-11

**Authors:** V. Heurtier, G. Marret, C. Lamy, A. Hamza, M.H. Diallo, L. Ahmanache, S. Vacher, A. Schnitzler, L. Courtois, E. Jeannot, J. Klijanienko, Z. El Beaino, J. Gal, M. Ledru, C. Martinat, J. Martin, O. Choussy, A. Dubray-Vautrin, M. Lesnik, W. Ghanem, R. Taouachi, N. Badois, C. Nhy, E. Borcoman, J. Flavius, A. Kadi, F. Berger, F. Servant, M. Kamal, I. Bièche, C. Le Tourneau

**Affiliations:** 1 Department of Genetics, Institut Curie, PSL Research University, Paris, France; 2 Computational Oncology, PSL Research University, Mines Paris Tech, INSERM U1331, Paris, France; 3 Department of Drug Development and Innovation (D3i), Institut Curie, Paris-Saclay University, Paris, France; 4 Department of Biometry, Institut Curie, Saint-Cloud, France; 5 Department of Pathology, Institut Curie, Paris and Saint-Cloud, France; 6 University Côte d’Azur, Centre Antoine Lacassagne, Epidemiology and Biostatistics Department, Nice, France; 7 Department of Oncologic Surgery, Institut Curie, PSL Research University, Paris, France; 8 Cordeliers Research Center, Sorbonne Université, INSERM, Université Paris Cité, Inflammation, Complement, and Cancer Team, Paris, France; 9 Radiology Department, Institut Curie Hospital, Paris, France; 10 IHU PRISM National PRecISion Medicine Center in Oncology, Gustave Roussy, Villejuif, France; 11 Faculty of Pharmaceutical and Biological Sciences, Paris Cité University, INSERM U1016, Paris, France

**Keywords:** head and neck squamous-cell carcinoma (HNSCC), intratumoral heterogeneity, molecular oncology

## Abstract

**Background:**

Intratumoral heterogeneity (ITH) is thought to contribute to tumour evolution and treatment resistance but its biological and clinical significance in localised head and neck squamous-cell carcinoma (HNSCC) remains incompletely understood.

**Patients and methods:**

In the prospective SCANDARE study, we analysed 87 patients with resectable HNSCC treated with upfront surgery. Two to five spatially distinct tumour regions per patient underwent pathological evaluation, targeted DNA sequencing, and bulk RNA sequencing. Genomic ITH (gITH) was quantified using clonal deconvolution and Shannon diversity indices, whereas transcriptional heterogeneity (tITH) was assessed using the intratumour expression distance metric. Associations between ITH, molecular features, tumour microenvironment composition, and clinical outcomes were explored using multivariable statistical models.

**Results:**

Pathology-based spatial heterogeneity showed limited prognostic value. gITH was common, with 37% of tumours displaying regionally heterogeneous pathogenic variants, including spatially actionable alterations in 10% of patients. In an initial multivariable Cox model, higher gITH was associated with shorter disease-free survival. However, after Ridge-penalised modelling and bootstrap internal validation, the effect size was attenuated [corrected hazard ratio 1.42, 95% confidence interval (CI) 0.91-2.75]. The overall model retained moderate discriminative performance (optimism-corrected C-index 0.69, 95% CI 0.59-0.79). gITH was associated with tumour cellularity, reduced estimated endothelial cell infiltration, and alterations in KMT2C and PIK3CA. tITH differed according to human papillomavirus (HPV) status, with lower tITH in HPV-positive tumours, and was associated with distinct biological pathways and genomic alterations. Genomic and tITH were not correlated.

**Conclusions:**

This prospective multiregion study provides a comprehensive characterisation of genomic and tITH in localised HNSCC. Our findings highlight substantial spatial molecular diversity within primary tumours and suggest potential associations between heterogeneity, tumour biology, and clinical outcome that warrant validation in independent cohorts.

## Highlights

• Prospective multiregion DNA and RNA-seq reveals extensive ITH in localised HNSCC.
• gITH is frequent and captures biologically distinct tumour features.
• Spatially heterogeneous actionable alterations are identified in 10% of patients.
• Transcriptional heterogeneity differs by HPV status and reflects distinct tumour biology.

## Introduction

Head and neck squamous-cell carcinoma (HNSCC) is the most common malignancy arising from the upper aerodigestive tract. Despite advancements in clinical management, a substantial proportion of patients with localised HNSCC experience locoregional or distant relapse.^1^, ^2^, ^3^, ^4^ Current prognostic indicators—primarily based on clinical and histopathological features, including human papillomavirus (HPV) status—often fall short of accurately predicting HNSCC recurrence.^5^ This underscores the need for improved risk stratification and personalised therapeutic strategies.

Tumour evolution is shaped by a complex interplay of genomic alterations and transcriptional reprogramming, leading to the coexistence of diverse cellular phenotypes within the same tumour. Next-generation sequencing technologies combined with multiregion sequencing have enabled a comprehensive characterisation of both genomic and transcriptomic heterogeneity, offering valuable insights into the spatial architecture of molecular diversity within tumours.^6^^,^^7^ This detailed understanding of intratumoral heterogeneity (ITH) may improve predictions of tumour evolution and relapse.

Genomic ITH (gITH) has been established as a remarkable predictor of clinical outcomes in several cancer types.^8^^,^^9^ Emerging evidence suggests that transcriptional heterogeneity (tITH) may also play a major role in determining prognosis.^6^ A few pioneering studies have highlighted the prognostic value of gITH in localised HNSCC estimated through the mutant allele tumour heterogeneity score, defined as the median-normalised width of the distribution of mutant allele fractions.^10^, ^11^, ^12^, ^13^ However, the clinical impact of these investigations has been limited to retrospective analyses and the need for available whole-exome sequencing carried out on a single tumour sample. Consequently, little is known about the spatial patterns of ITH in HNSCC—particularly at the transcriptomic level—and how they relate to gITH and patient outcomes.

In this study, we present a comprehensive analysis of spatial phenotypic and molecular ITH in a prospective cohort of 87 patients with nonmetastatic, resectable HNSCC enrolled in the SCANDARE prospective biobanking study (NCT03017573). Phenotypic ITH was assessed through histological differentiation patterns and correlated with relapse risk. We further carried out targeted DNA sequencing (DNA-seq) and bulk RNA sequencing (RNA-seq) across multiple tumour regions to evaluate gITH and tITH. The prognostic significance of these layers of heterogeneity was examined and genes associated with molecular ITH were identified. This work provides an integrated view of spatial tumour heterogeneity in localised HNSCC and identifies biological features associated with genomic and transcriptional diversification.

## Patients and methods

### Study design and patients

SCANDARE (NCT03017573) is a prospective biobanking study enrolling patients with nonmetastatic, resectable HNSCC treated with upfront curative-intent surgery. The present analysis includes 87 patients operated between 2017 and 2019 at Institut Curie. All patients provided written informed consent. Clinical data, treatments, and outcomes were prospectively collected.

### Multiregional tumour sampling and pathological assessment

For each tumour, a senior pathologist (J.K.) selected between one and five spatially distinct regions based on divergent differentiation patterns. Tumour cellularity and differentiation status (well differentiated compared with poorly differentiated) were assessed on hematoxylin–eosin-stained sections. A pathology-based spatial heterogeneity classification was defined a priori.

### DNA and RNA sequencing

Selected tumour regions underwent targeted DNA-seq using a 571-gene pan-cancer panel and bulk RNA-seq. Sequencing was carried out on formalin-fixed paraffin-embedded (FFPE) samples following standardised quality control criteria. HPV status was determined by PCR-based genotyping.

### Genomic and transcriptional heterogeneity assessment

gITH was quantified using clonal deconvolution and Shannon diversity indices, retaining the maximum gITH value per tumour as representative of peak clonal diversity. tITH was assessed using the intratumour expression distance (I-TED) metric based on pairwise comparisons between tumour regions, computed separately for HPV-positive and HPV-negative tumours.

### Statistical analysis

Associations between heterogeneity metrics, clinicopathological variables, and tumour microenvironment features were assessed using nonparametric tests and correlation analyses. Disease-free survival (DFS) and overall survival (OS) were estimated using Kaplan–Meier methods and compared using multivariable Cox proportional hazards models adjusted for established prognostic factors. Ridge-penalised Cox regression and double-bootstrap procedure were used to mitigate the risk of overfitting and for optimism correction.

## Results

### Study design

**Figure 1:**
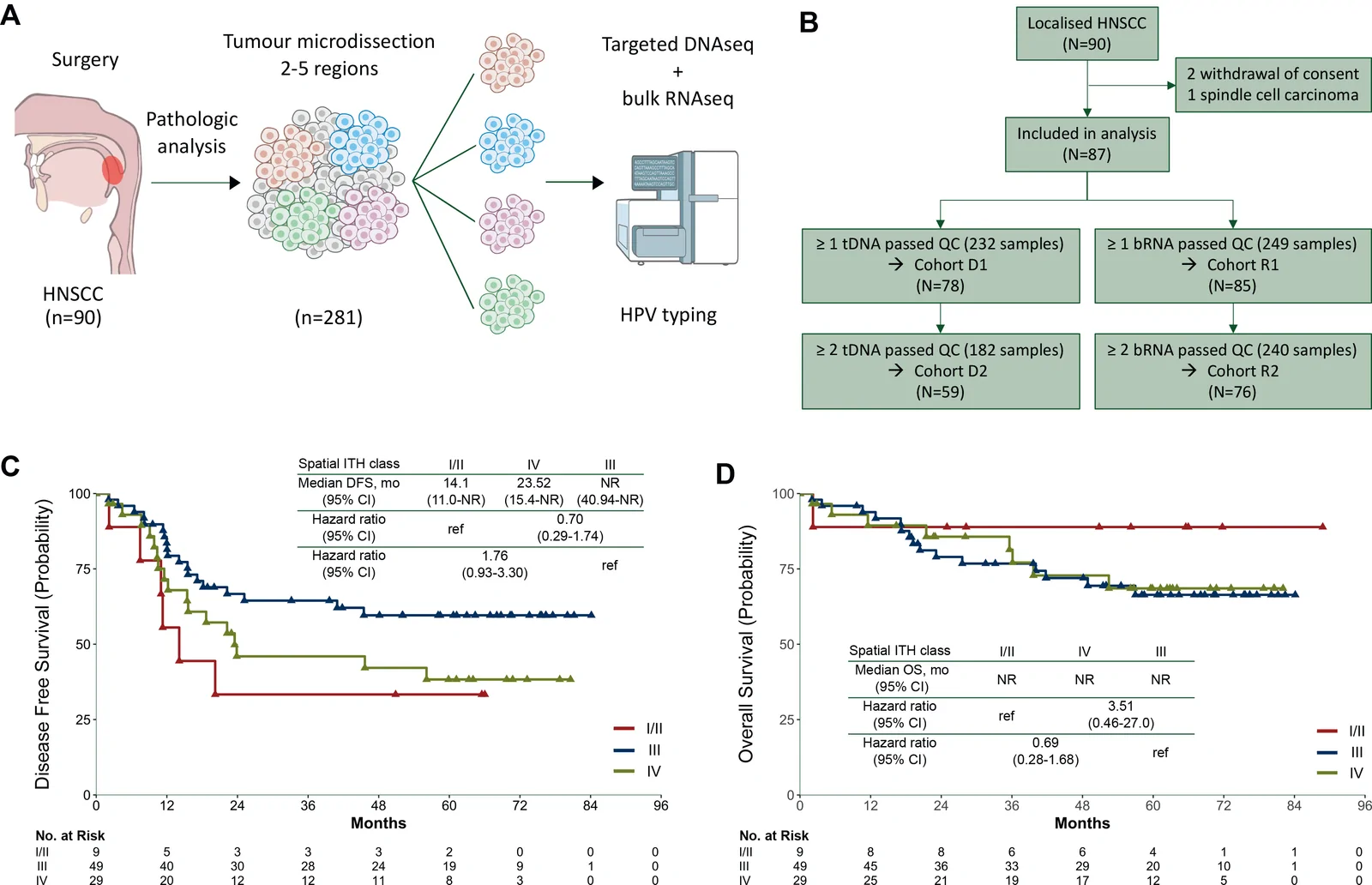
**Study design and pathological heterogeneity assessment.** (A) Schematic overview of the study design. Tumours from 90 patients with resectable HNSCC from the biobanking SCANDARE clinical study were microdissected into spatially distinct regions (*n* = 281) and profiled by targeted DNA-seq, RNA-seq, and HPV typing. (B) Flowchart of patient inclusion and sequencing. Targeted DNA-seq was carried out on ≥1 region in 78 patients (D1 cohort, 232 regions) and on ≥2 regions in 59 patients (D2 cohort, 220 regions). Bulk RNA-seq was carried out on ≥1 region in 85 patients (R1 cohort, 249 regions) and on ≥2 regions in 76 patients (R2 cohort, 240 regions). (C) Kaplan–Meier curve for DFS stratified by spatial ITH. (D) Kaplan–Meier curve for OS stratified by spatial ITH. The HRs and 95% CIs displayed correspond to the adjusted estimates from the multivariate Cox regression model. Variables included in the model are the following: sex, tumour and nodal stage, age group, HPV status, and spatial ITH class. RNA-seq, RNA sequencing; CI, confidence interval; DFS, disease-free survival; DNA-seq, DNA sequencing; HNSCC, head and neck squamous-cell carcinoma; HPV, human papillomavirus; HRs, hazard ratios; ITH, intratumoral heterogeneity; NR, not reached; OS, overall survival; QC, quality control; ref, reference; tDNA, targeted DNA-seq.

The SCANDARE clinical trial is a prospective biobanking study (NCT03017573, registration date: 9 January 2017) which recruited patients with nonmetastatic resectable HNSCC treated with upfront curative-intent surgery with or without adjuvant treatment after standard-of-care management. Informed written consent was given by all participants. FFPE tumour tissue from diagnostic surgery was sampled in two to five spatially distinct regions for 90 patients. Two patients withdrew consent and one patient diagnosed with a rare type of HNSCC (spindle cell carcinoma) was excluded from the analysis. Clinical data are available for the remaining 87 patients, including participant and tumour characteristics, received treatments and cancer outcomes and survival (Table 1). After a median follow-up of 63 months with a range from 2.9 months (among censored patients) to 88.8 months (overall), the number of relapses was 37/87 (43%). The percentage of HPV-positive tumour was 26% (23/87), mainly represented by tumours from the oropharynx (13/28). To investigate spatial phenotypic ITH, a single senior pathologist (Z.E.B.) selected one to five phenotypically dissimilar regions per tumour based on differentiation patterns for subsequent targeted DNA-seq and RNA-seq, totalling 281 tumour regions (Figure 1A). This pathologist was blinded to clinical data at the time of this assessment. We successfully carried out DNA-seq on at least 1 tumour region for 78 patients (D1 cohort, 232 tumour regions), and on at least 2 tumour regions for 59 patients (D2 cohort, 182 tumour regions). Similarly, RNA-seq was conducted on at least one tumour region for 85 patients (R1 cohort, 249 tumour regions), and on at least two tumour regions for 76 patients (R2 cohort, 240 tumour regions; Figure 1B).

**Table 1:** Patient and tumour characteristics with univariate log-rank analysis for DFS and OS

Variable		*N* = 87 (%)	DFS [HR (95% CI), *P*]	OS [HR (95% CI), *P*]
Age (years)				
	<65 (ref)	43 (49)		
	≥65	44 (51)	1.95 (1.05-3.63), 0.034	4.30 (1.70-10.9), 0.002
Sex				
	Female (ref)	29 (33)		
	Male	58 (67)	2.10 (1.03-4.27), 0.041	2.38 (0.89-6.39), 0.085
Tumour stage				
	T1	25 (29)		
	T2	26 (30)		
	T3	15 (17)		
	T4	21 (24)		
	T3-4 versus T1-2 (ref)	51 (59) versus 36 (41)	2.20 (1.20-4.05), 0.011	2.02 (0.90-4.53), 0.089
Nodal stage				
	N0	45 (52)		
	N1	22 (25)		
	N2	3 (3.4)		
	N2a	3 (3.4)		
	N2b	8 (9.2)		
	N2c	4 (4.6)		
	N3b	2 (2.3)		
	N1-3 versus N0 (ref)	45 (52) versus 42 (48)	1.65 (0.90-3.04), 0.105	1.97 (0.86-4.50), 0.108
Metastasis stage				
	M0	87 (100)		
	M1	0 (0)	—	—
Tumour differentiation				
	Moderately/well (ref)	78 (90)		
	Undifferentiated/poorly	9 (10)	0.57 (0.18-1.86), 0.354	0.67 (0.16-2.83), 0.582
Localisation				
	Hypopharynx (ref)	5 (5.7)		
	Larynx	9 (10)	0.51 (0.11-2.27), 0.375	0.59 (0.10-3.55), 0.567
	Oral cavity	45 (52)	0.47 (0.14-1.60), 0.229	0.54 (0.12-2.40), 0.420
	Oropharynx	28 (32)	0.68 (0.20-2.36), 0.545	0.38 (0.08-1.87), 0.232
Alcohol consumption history				
	NA	1 (1.1)		
	Yes (ref)	39 (45)		
	No	47 (54)	1.24 (0.67-2.29), 0.495	1.11 (0.50-2.48), 0.796
Tobacco consumption history				
	NA	1 (1.1)		
	Yes (ref)	47 (54)		
	No	39 (45)	1.27 (0.69-2.36), 0.446	1.36 (0.60-3.07), 0.455
Tumour HPV status				
	Negative (ref)	64 (74)		
	Positive	23 (26)	0.60 (0.28-1.29), 0.191	0.48 (0.16-1.40), 0.176
Adjuvant chemotherapy				
	Yes (ref)	18 (21)		
	No	69 (79)	0.69 (0.31-1.56), 0.373	0.94 (0.35-2.52), 0.905
Adjuvant radiotherapy				
	Yes (ref)	51 (59)		
	No	36 (41)	0.84 (0.45-1.54), 0.570	1.26 (0.54-2.96), 0.588
Recurrence				
	Yes	37 (43)		
	No	50 (57)	—	—

Log-rank analysis from univariate survival analysis for DFS and OS. HRs are shown with 95% CI. Ref categories are indicated in the table. Continuous or multilevel variables were categorised for analysis as follows: tumour stage: T3-4 versus T1–2 (Ref). Nodal stage: N1–3 versus N0 (Ref). Other variables retained their original categories.

CI, confidence interval; DFS, disease-free survival; HPV, human papillomavirus; HRs, hazard ratios; NA, not available; OS, overall survival; ref, reference.

### Spatial pathological ITH in HNSCC is an imperfect predictor of relapse

A key parameter of morphological assessment in HNSCC is determining the tumour’s degree of cell differentiation based on keratinisation, mitotic activity, cancer cell pleomorphism, pattern of invasion, and surrounding tissue response.^14^ Although regions with distinct differentiation grades can be found within the same tumour, pathological reports usually take into consideration only the most abundant one, leading to a loss of information regarding ITH. We sought to evaluate this phenotypic ITH within the SCANDARE cohort and examine its association with cancer relapse. For increased reproducibility and feasibility, we adopted a binary classification of differentiation (well- or poorly differentiated) for subsequent analyses. The mean percentage of well- and poorly-differentiated regions in each tumour was assessed by a senior pathologist (J.K.). We defined a priori four tumour classes: homogeneous tumours with either >80% of well-differentiated (I) or poorly-differentiated cells (II), and heterogeneous tumours, categorised according to a predominance of either well-differentiated (III) or undifferentiated cells (IV).

Most tumours in our cohort exhibited a heterogeneous differentiation pattern, with only two class I and seven class II tumours identified. Consequently, our predefined classification primarily distinguished tumours based on their predominant differentiation status. The only patient characteristic significantly associated with differentiation class was HPV status, with a higher prevalence of IV tumours among HPV-negative tumours (*P =* 0.046; Supplementary Figure S1A, available at https://doi.org/10.1016/j.esmoop.2026.108367). There was also a nonsignificant trend linking class I/II tumours to patient smoking history (*P =* 0.056; Supplementary Figure S1B, available at https://doi.org/10.1016/j.esmoop.2026.108367).

No statistically significant association was observed between differentiation classes and DFS or OS, either across the four classes or when comparing heterogeneous versus homogeneous tumours (Figure 1C and D). Although class III tumours showed numerically longer DFS, confidence intervals (CIs) were wide and results remained nonsignificant. Likewise, the numerically longer DFS observed for class IV compared with the homogeneous classes should be interpreted cautiously, as the pathological ITH classification reflects spatial differentiation heterogeneity rather than tumour stage or overall tumour aggressiveness and the homogeneous classes included only a small number of patients.

These findings suggest that histological heterogeneity alone may not robustly predict outcome in this cohort, supporting further investigation of the molecular features underlying tumour heterogeneity.

### The landscape of genetic ITH at the single-variant scale

**Figure 2:**
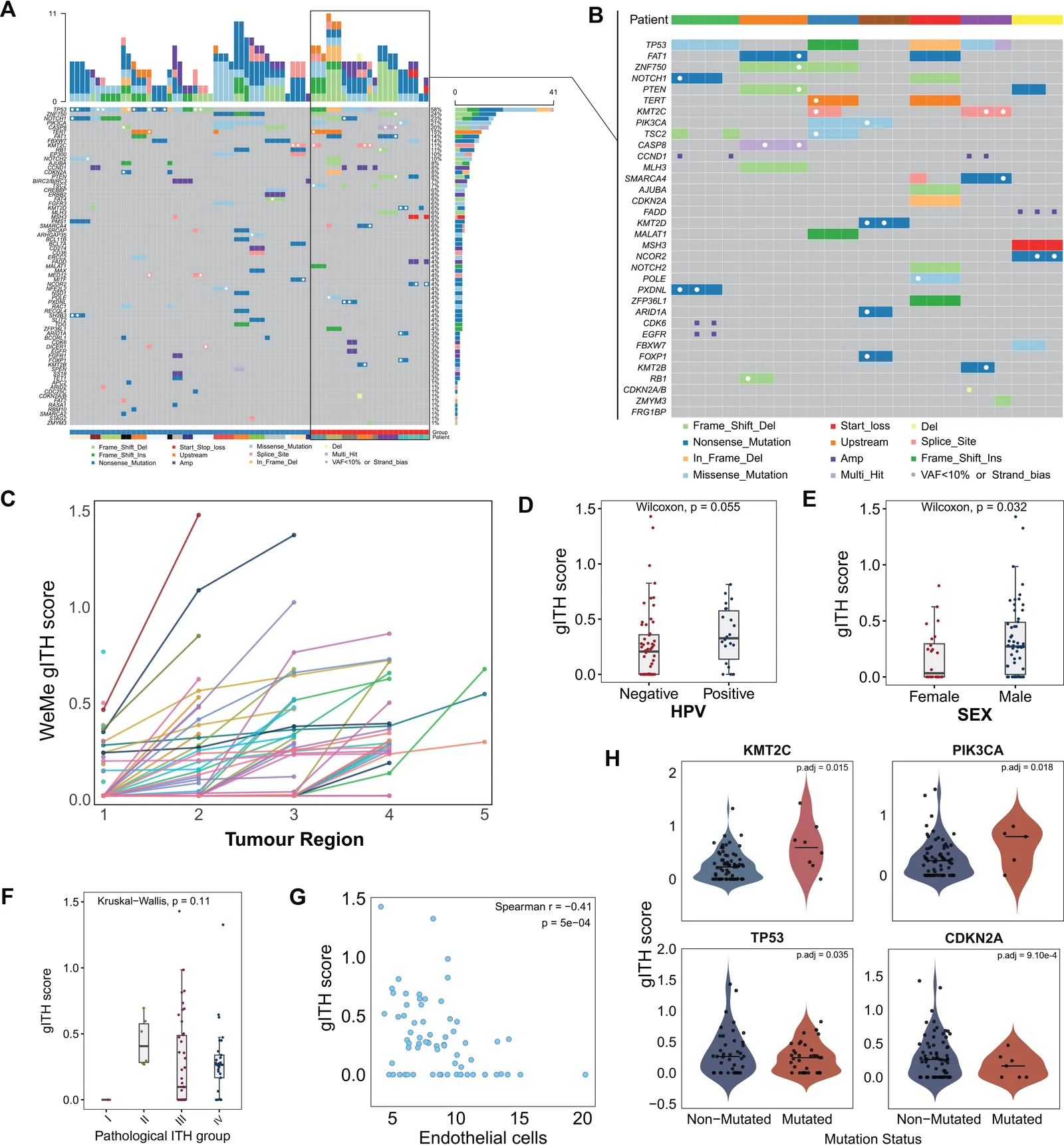
**gITH analysis.** (A) Oncoplot showing the mutational landscape of 22 patients (37% of the D2 cohort) who harboured regionally heterogeneous PVs across spatially distinct tumour regions. Only variants with an allelic frequency > 10% were considered validated. Heterogeneous variants with VAF < 10% and strand bias are indicated by a white dot as additional information. Patients harbouring heterogeneous ‘actionable’ variants are coloured in red on the group legend below. (B) Oncoplot of seven patients with regionally heterogeneous PVs in clinically actionable genes, as defined by the ESMO Scale for Clinical Actionability of molecular Targets (tiers I-V) or the OncoKB database (levels 1-4).^17^, ^18^, ^19^ (C) Distribution of WeMe gITH scores across patients (*n* = 78) according to the number of tumour regions sequenced. Each line represents a single patient, with individual trajectories shown in distinct colours. Lines connect the available measurements for each patient across its distinct tumour regions. Isolated points correspond to patients with only one available measurement of gITH. (D) gITH score distribution stratified by HPV status. (E) gITH score distribution stratified by sex. (F) gITH score distribution according to pathological ITH classification. (G) gITH score distribution plotted against endothelial cell enrichment scores, derived from RNA-seq data using MCPcounter.^20^ (H) gITH score distribution stratified by mutational status of *CDKN2A*, *KMT2C*, *PIK3CA*, and *TP53* genes (Wilcoxon test, Benjamini–Hochberg adjusted *P* value). Amp, amplification; Del, deletion; gITH, genomic ITH; HPV, human papillomavirus; Ins, insertion; PVs, pathogenic variants; VAF, variant allele frequency.

Genetic divergence within a tumour leads to the emergence of coexisting clonal populations, each defined by shared ancestral mutations and region-specific variants acquired during tumour evolution.^15^ This diversity underpins genetic ITH. To assess this phenomenon, we first analysed the distribution of pathogenic variants (PVs)—used here as a qualitative proxy of multisample ITH—across spatially distinct tumour regions in the D2 cohort using DNA-seq. Overall, genomic profiles were consistent with previous molecular studies of localised HNSCC (Supplementary Table S1, available at https://doi.org/10.1016/j.esmoop.2026.108367).^16^ We found that 37% (22/59) of the tumours exhibited heterogeneous PVs across their respective multiple regions (Figure 2A) among the 571 sequenced genes of our panel. Next, we assessed whether the heterogeneity of these PVs may have influenced the therapeutical strategy of the respective tumours in another clinical context. We found that in six of these patients (10%, 6/59), the heterogeneous PVs were actionable (Figure 2B) according to the ESMO Scale for Clinical Actionability of molecular Targets (tiers I-V) or OncoKB database (levels 1-4)—i.e. their detection could influence eligibility for targeted therapy.^17^, ^18^, ^19^ Consequently, ITH could have directly impacted the clinical management of these patients in a clinical setting where treatment decisions are based on the results of a single region analysis.

### The landscape of gITH at clonal scale

Subclonal deconvolution enables the dissection of a tumour’s clonal composition from bulk sequencing data. We applied four widely validated clonal deconvolution algorithms, alongside the consensus WeMe method, to infer clonal diversity in the D1 cohort.^21^, ^22^, ^23^, ^24^, ^25^ These algorithms primarily output the number and cell fractions of tumour subclones identified in each sample, from which we calculated the Shannon diversity index as a quantitative measure of single-sample gITH. In total, 232 tumour regions were analysed (D1 cohort) and 230 were successfully processed by clonal deconvolution pipelines, with a median of 3 regions per patient (range: 1-5; Supplementary Figure S2A, available at https://doi.org/10.1016/j.esmoop.2026.108367). We found no significant difference in clonal heterogeneity between well-differentiated and poorly-differentiated regions, as assessed phenotypically by our pathologist (J.K.) (Supplementary Figure S2B, available at https://doi.org/10.1016/j.esmoop.2026.108367). As expected, we found a positive correlation between tumour sample cellularity and genomic heterogeneity detection (Supplementary Figure S2C, available at https://doi.org/10.1016/j.esmoop.2026.108367). Figure 2C illustrates the dynamics of the gITH score (WeMe) across tumour regions, revealing that spatially distinct tumour regions can exhibit strikingly different clonal diversity. To derive a patient-level measure of gITH, we used the maximum gITH value observed among all sampled regions from a given tumour (max-gITH). This approach was chosen to capture the highest level of subclonal diversity detected within a tumour, under the hypothesis that biologically relevant subclonal populations may be spatially restricted and therefore not reflected by average heterogeneity measures. This metric emphasises regions with the greatest clonal diversity rather than overall tumour-wide heterogeneity and should therefore be interpreted as a measure of peak detected heterogeneity. Overall, all tools produced consistent scores that were positively correlated (Supplementary Figure S2D, available at https://doi.org/10.1016/j.esmoop.2026.108367). The max-gITH scores obtained from the different methods showed a linear relationship with the number of detected clones (Supplementary Figure S2E, available at https://doi.org/10.1016/j.esmoop.2026.108367). Therefore, we focused our analysis on the consensus WeMe score and from this point forward, all mentions of gITH score refer to this maximum value (max-gITH) of the WeMe score per patient (median: 0.25, min–max: 0-1.43). The median number of detected clones across the 78 tumours was 2 (range: 1-4). The gITH score of a given patient was weakly correlated with the number of samples studied (Spearman rho = 0.29, *P =* 1.45e−05; Supplementary Figure S2F, available at https://doi.org/10.1016/j.esmoop.2026.108367). We explored correlations between clonal diversity and patient/tumour characteristics. HPV-negative tumours tended to have a higher median gITH score, although the difference did not reach statistical significance (*P =* 0.055; Figure 2D). Among clinical characteristics, sex was the only factor significantly influencing clonal diversity, with male patients showing a higher gITH score overall (*P =* 0.032; Figure 2E). Neither tumour localisation, T or N stage, patient age, nor alcohol or tobacco exposure appeared to affect clonal heterogeneity in our cohort (Supplementary Figure S2G, available at https://doi.org/10.1016/j.esmoop.2026.108367). Interestingly, when correlating gITH scores with our previous phenotypic classification, we found that type II tumours (homogeneous, poorly differentiated) exhibited high gITH despite their phenotypic uniformity (Figure 2F). Moreover, gITH tended to reflect the overall fraction of undifferentiated cells across the entire tumour (Figure 2F) rather than mirroring the differentiation status of individual tumour subregions (Supplementary Figure S2C, available at https://doi.org/10.1016/j.esmoop.2026.108367).

To further investigate the link between tumour microenvironment and genetic ITH, we applied MCPcounter-based deconvolution on pooled tumour-level RNA-seq data.^20^ This revealed a moderate inverse correlation between clonal heterogeneity and endothelial cell infiltration (Spearman rho = −0.41, *P <* 0.01; Figure 2G) but no association with lymphoid or innate immune infiltration (Supplementary Figure 2H, available at https://doi.org/10.1016/j.esmoop.2026.108367). Given the use of bulk RNA-based deconvolution, these findings should be interpreted cautiously, as tumour purity and stromal content may influence both inferred microenvironment composition and heterogeneity estimates.

Finally, we investigated potential driver variants of ITH by assessing the correlation between gITH and gene alterations. Tumours harbouring *KMT2C* and *PIK3CA* mutations exhibited higher gITH, whereas *TP53*- and *CDKN2A*-mutated tumours tended to have lower clonal diversity (Figure 2H).

In summary, genetic clonal heterogeneity, as inferred from PV differences, was associated with tumour cell purity, patient sex, reduced vascularisation, and distinct gene alterations.

### Spatial genetic ITH and clinical outcomes

We next explored the association between tumour clonal heterogeneity, estimated using the gITH score, and clinical outcome in the D1 cohort. Patients were stratified into gITH quartiles (low, intermediate-low, intermediate-high, and high gITH) with most patients in the lowest quartile exhibiting scores close to zero (Supplementary Figure S3A, available at https://doi.org/10.1016/j.esmoop.2026.108367).

**Figure 3:**
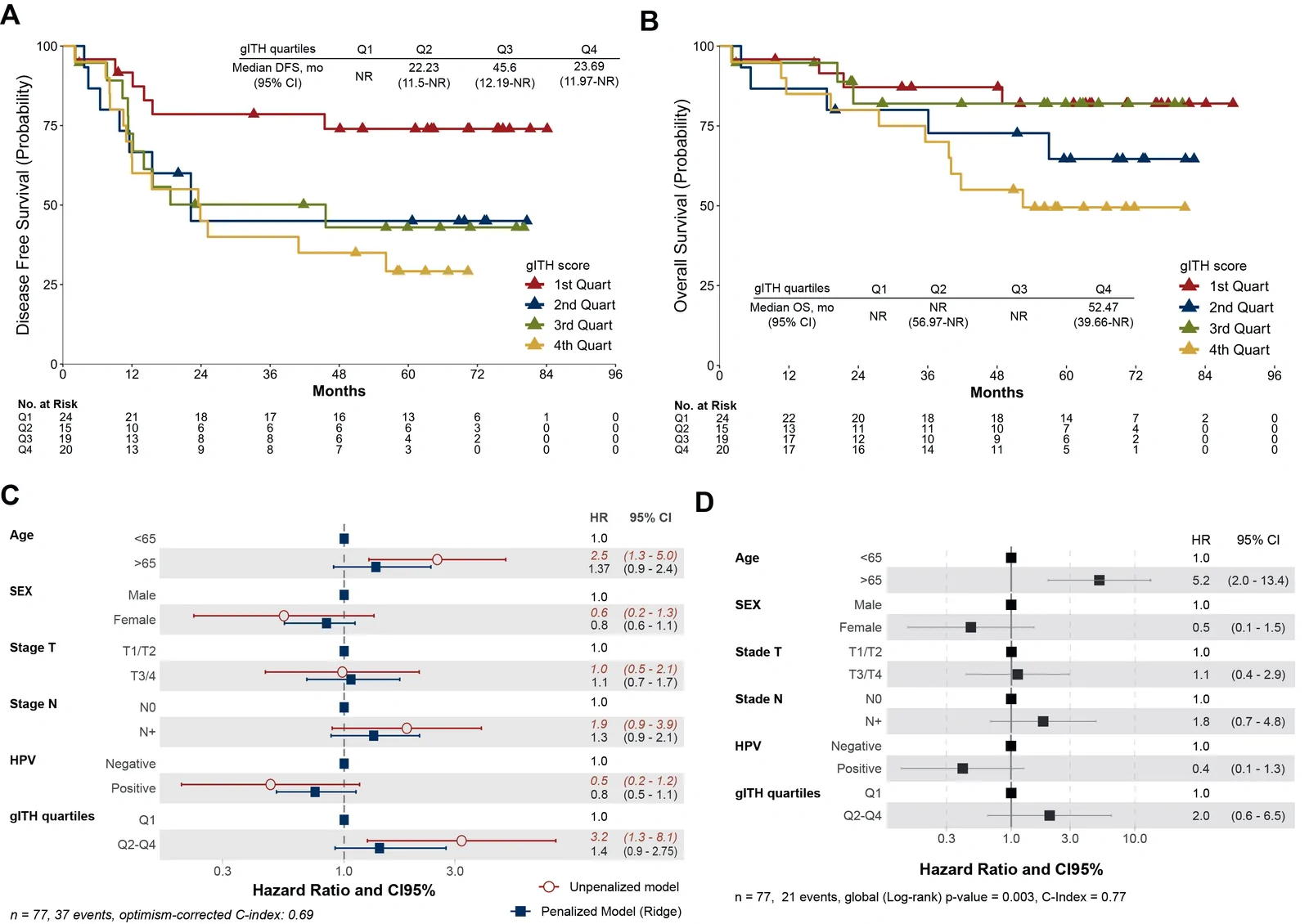
**Genetic ITH is an independent prognostic factor in HNSCC.** (A) Kaplan–Meier curve for DFS across quartiles (Q1-Q4) of gITH, showing shorter DFS in patients with intermediate-, low-, to high gITH. The distribution of patients across quartiles is not perfectly balanced due to the asymmetric distribution of gITH scores (see Supplementary Figure S3A, available at https://doi.org/10.1016/j.esmoop.2026.108367). (B) OS according to gITH quartiles. (C) Multivariable analysis of DFS including gITH and clinical covariates. Forest plot showing HRs estimated from the conventional multivariable Cox proportional hazards model (in red) and from the Ridge-penalised Cox model after bootstrap-based optimism correction (in blue). gITH was included as a categorical variable (Q2-Q4 versus Q1). Error bars represent 95% CIs. (D) Multivariate Cox proportional hazards model (without optimism correction) for OS, adjusted for clinical covariates with gITH included as a categorical variable (Q2-Q4 versus Q1). CI, confidence interval; DFS, disease-free survival; gITH, genomic ITH; HNSCC, head and neck squamous-cell carcinoma; HRs, hazard ratios; NR, not reached; OS, overall survival; Quart, quartile.

In a multivariate Cox model including T and N stage, age, HPV status, and sex, intermediate-low to high gITH tumours (second-fourth quartiles) were initially associated with shorter DFS compared with gITH-low tumours [median 23.5 versus not reached (NR) months, hazard ratio (HR) 3.29, 95% CI 1.30-8.3, *P* = 0.012] (Figure 3A and Supplementary Figure S3, available at https://doi.org/10.1016/j.esmoop.2026.108367). However, given the limited number of events and the potential risk of model overfitting, we subsequently applied a Ridge-penalised Cox regression combined with double-bootstrap internal validation. After optimism correction, the estimated effect size was substantially attenuated (corrected HR 1.42, 95% CI 0.91-2.75), the CI crossing the null value, indicating that the prognostic impact of gITH could not be established robustly in this cohort. Nevertheless, the overall multivariable model retained moderate discriminative performance, with an optimism-corrected C-index of 0.69 (95% CI 0.59-0.79), suggesting that tumour heterogeneity may still capture clinically relevant biological variability. None of the other prognostic variables included in the model (T and N stage, age, HPV status, and sex) showed significant corrected associations with DFS (Figure 3B). We also explored potential associations between gITH-low status and clinicopathological features (Figure 3C and D). The only significant association was an enrichment of class III tumours within the gITH-low group (75% versus 44.4%, *P* = 0.049; Supplementary Figure S3B, available at https://doi.org/10.1016/j.esmoop.2026.108367). Overall, these findings support a possible association between molecular ITH and clinical outcome, although the magnitude and robustness of this effect remain uncertain and require validation in larger independent cohorts.

### The landscape of tITH

**Figure 4:**
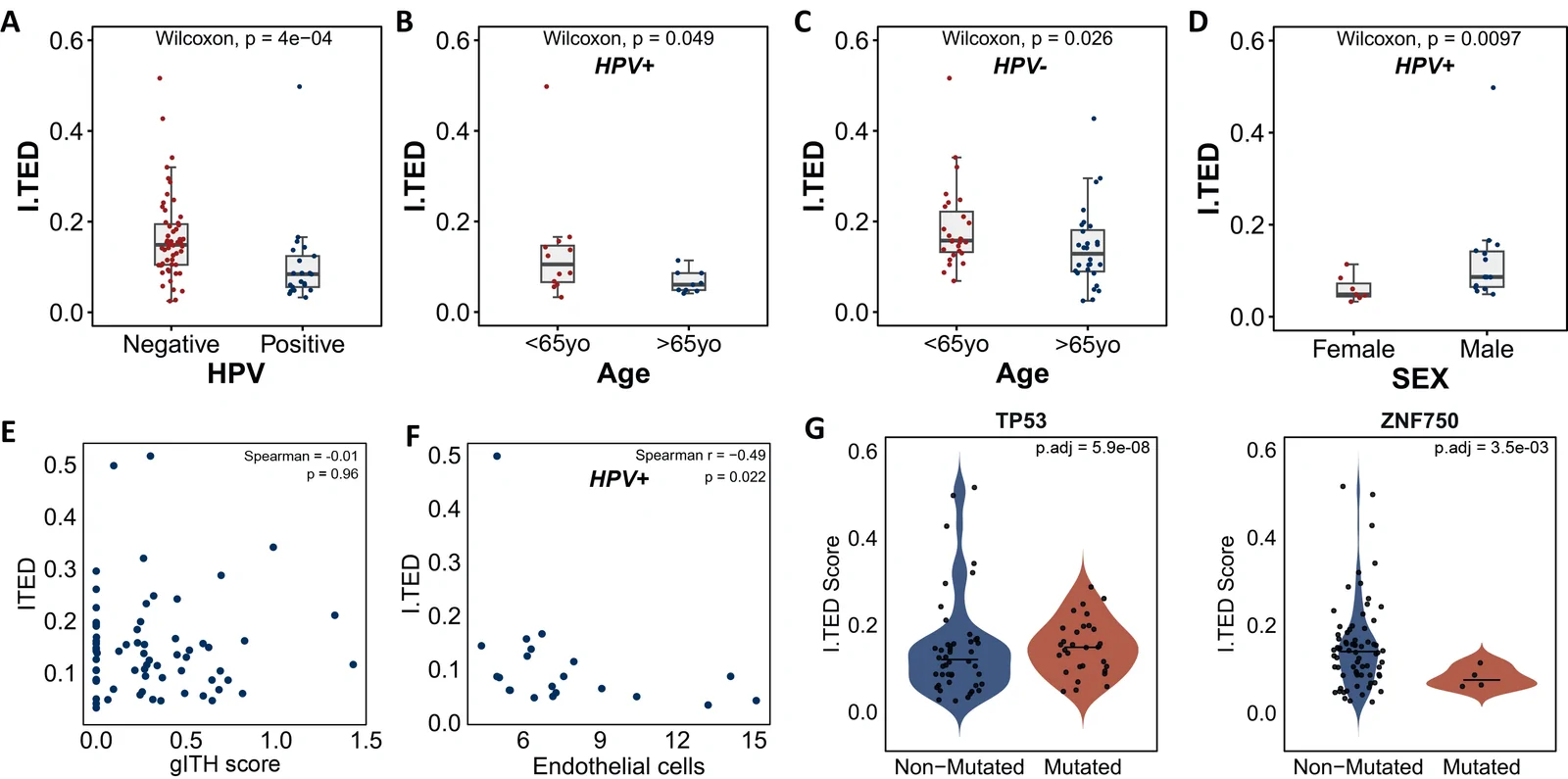
**Transcriptomic ITH and tumour subtypes.** (A) Distribution of the I-TED score stratified by HPV status. (B) Distribution of the I-TED score stratified by age category (≤65 versus >65 years) among patients who are HPV-positive. (C) Distribution of the I-TED score stratified by age category (≤65 versus >65 years) among patients who are HPV-negative. (D) Distribution of the I-TED score stratified by sex among patients who are HPV-positive. (E) Distribution of the I-TED score according to gITH score. (F) I-TED score distribution plotted against endothelial cell enrichment scores, derived from RNA-seq data using MCPcounter,^20^ in patients who are HPV-positive. (G) I-TED score distribution stratified by mutational status of *TP53* and *ZNF750* genes (Wilcoxon test, Benjamini–Hochberg adjusted *P* value). gITH, genomic ITH; HPV, human papillomavirus; I-TED, intratumour expression distance.

Recent evidence highlights the role in ITH of gene-expression variability, which may arise from gene mutations or copy number alterations, epigenomic modifications, and posttranscriptional regulation.^6^ In total, 240 tumour regions from 76 patients in the R2 cohort were successfully sequenced using RNA-seq and analysed, with a median of 3 regions per patient (range: 2-5). To estimate tITH, we analysed RNA-seq data from multiple tumour regions within the R2 cohort using the I-TED metric.^6^ I-TED translates tITH into a numerical score—I-TED—by performing pairwise comparisons between tumour regions. The score is computed based on correlation distances between samples from the same tumour, derived from the expression of the 500 most variable genes in the whole dataset. Given that HPV-induced and HPV-independent tumours exhibit distinct transcriptional profiles,^26^ we expected the most variable genes to differ between these two tumour types. Indeed, when identifying the top 500 most variable genes separately for each group, only 294 genes were shared between them. As HPV-independent tumours were more prevalent in our cohort, pooling all tumours together without accounting for HPV status would bias the selection of the most variable genes towards those differing in HPV-independent samples. This could lead to a loss of critical variability in HPV-induced tumours. To mitigate this bias, we calculated I-TED scores separately for HPV-positive and HPV-negative tumours, using the top 500 most variable genes within each category (Supplementary Figure S4A, available at https://doi.org/10.1016/j.esmoop.2026.108367). Interestingly, HPV-positive tumours exhibited significantly lower I-TED scores than HPV-negative tumours (*P =* 4e−04; Figure 4A). This finding goes along with the fact that HPV-positive tumours are typically less altered at the genomic level, with fewer genome rearrangements and gene alterations.^27^ The strong oncogenic activity driven by HPV may also constrain tumour cells within a narrower transcriptional landscape. Despite a trend in patients who are HPV-negative, I-TED scores were not significantly correlated with either the mean or the maximum difference in tumour purity across samples from the same tumour (Supplementary Figure S4B and C, available at https://doi.org/10.1016/j.esmoop.2026.108367). We further explored potential associations between tITH and patient or tumour characteristics. I-TED scores were significantly higher in older patients (>65 years) in both HPV-positive and HPV-negative tumours (*P =* 0.049 and 0.026, respectively; Figure 4B and C). Additionally, male patients had significantly higher I-TED scores than female patients in HPV-positive tumours (*P =* 0.0097) but no such difference was observed in HPV-negative tumours (*P =* 0.34; Figure 4D and Supplementary Figure S4D, available at https://doi.org/10.1016/j.esmoop.2026.108367). No other significant associations were found between I-TED metrics and patient or tumour characteristics (Supplementary Figure S4D and E, available at https://doi.org/10.1016/j.esmoop.2026.108367). Notably, we observed no correlation between the gITH score and tITH metrics, consistent with previous findings^6^ (Figure 4E). We further investigated the link between tITH and the tumour microenvironment composition using MCPcounter.^20^ This analysis only revealed a weak but significantly negative association between I-TED and endothelial cell infiltration in HPV-positive tumours (Spearman rho = −0.49, *P =* 0.02; Figure 4F). However, no such association was observed between the I-TED score and any cell type of the tumour microenvironment in HPV-negative tumours (Supplementary Figure S4F and G, available at https://doi.org/10.1016/j.esmoop.2026.108367). As these analyses relied on bulk RNA-seq deconvolution, the observed associations may partly reflect variations in tumour purity or stromal content. Lastly, we examined potential driver variants influencing tITH by assessing the correlation between I-TED scores and gene alterations. We found two significant associations with *TP53*-mutated tumours exhibited higher I-TED scores whereas those harbouring *ZNF750* mutations had lower tITH (Figure 4G).

These findings suggest that HPV-positive tumours exhibit lower tITH, potentially due to their reduced genomic instability and the constraints imposed by HPV-driven oncogenesis. Moreover, tITH appears to be shaped by patient characteristics and specific genomic alterations in an HPV-dependent manner, highlighting distinct tumour biology driven by different oncogenic processes.

### Spatial tITH is not significantly associated with prognosis

**Figure 5:**
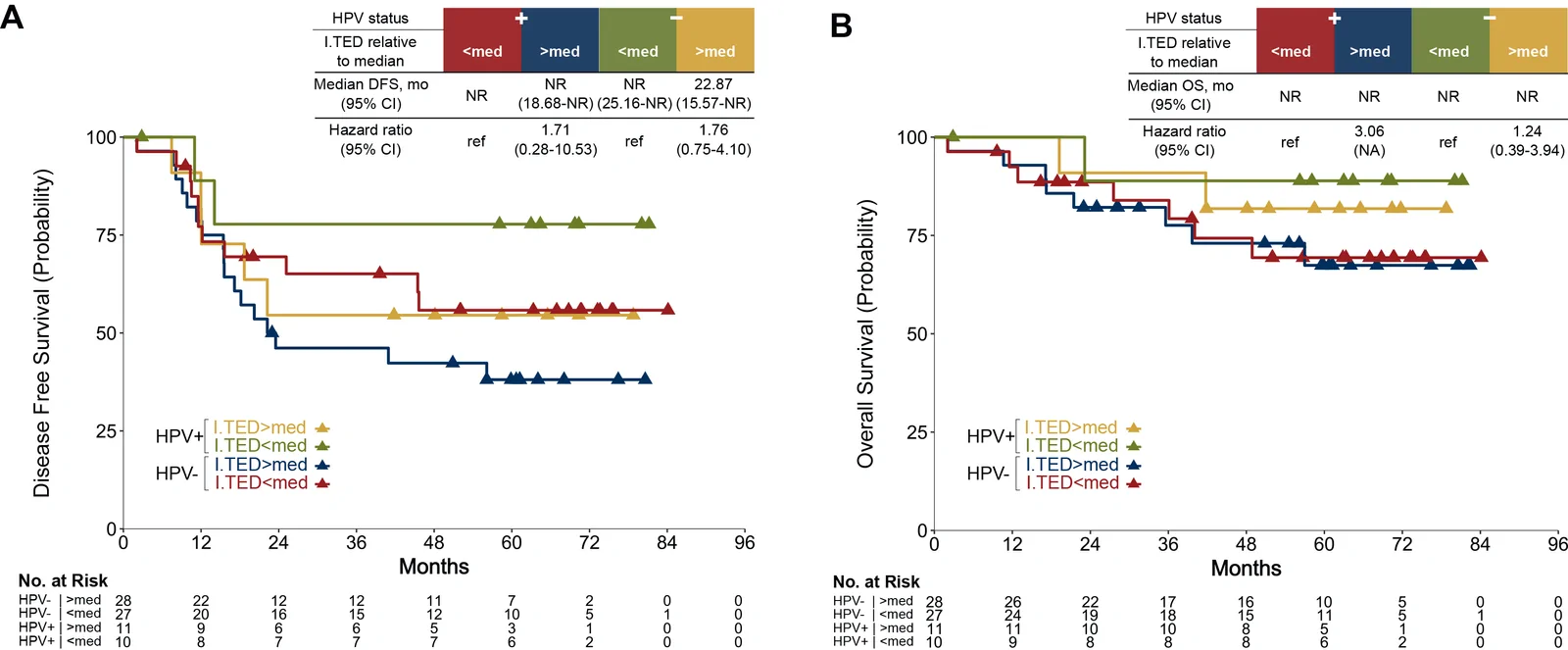
**Patient survival according to tITH analysis.** (A) Kaplan–Meier curves for DFS stratified by HPV status (positive versus negative) and I-TED score category (<median versus ≥ median). (B) Kaplan–Meier curves for OS stratified by HPV status (positive versus negative) and I-TED score category (< median versus ≥ median). The HRs and 95% CIs displayed correspond to the adjusted estimates from the multivariate Cox regression model. Variables included in the model are the following: sex, tumour and nodal stage, age group, HPV status, and I-TED score category (<median versus ≥median). CI, confidence interval; DFS, disease-free survival; HPV, human papillomavirus; HRs, hazard ratios; I-TED, intratumour expression distance; med, median; NR, not reached; OS, overall survival; ref, reference; tITH, transcriptional heterogeneity.

We analysed the prognosis of patients from the R2 cohort based on the tITH of their tumours, as estimated by the I-TED score. Patients were dichotomised at the median of the I-TED distribution (calculated separately for HPV-positive and HPV-negative tumours) according to the score distribution after a rough Gaussian shape (Supplementary Figure S5A and B, available at https://doi.org/10.1016/j.esmoop.2026.108367). No significant differences in DFS were observed between patients with I-TED scores above and below the median (HPV-positive: NR versus NR months, HR 1.71, 95% CI 0.28-10.53, *P* = 0.57; HPV-negative: 22.9 versus. NR months, HR = 1.76, 95% CI 0.75-4.10, *P* = 0.19; Figure 5A). Similarly, OS did not differ significantly between groups (HPV-positive: NR versus NR months, HR 3.06, 95% CI not estimable due to sparse data, *P* = 0.99; HPV-negative: NR versus NR months, HR 1.24, 95% CI 0.39-3.94, *P* = 0.23; Figure 5B).

In this limited cohort, tITH did not reach statistical significance for DFS or OS in either HPV-positive or HPV-negative tumours. These findings should be interpreted with caution owing to the small sample size and further studies in larger and independent cohorts are needed.

### Differential gene expression and tumour heterogeneity

We sought to identify genes whose expression correlates with either genomic or transcriptomic heterogeneity to uncover potential drivers of tumour heterogeneity. To this end, we carried out differential expression analysis using DESeq2,^28^ incorporating either gITH or tITH scores into linear models for each tumour from the R1 cohort. For each patient, RNA-seq data from all sampled regions were averaged to reduce the influence of regional variability in tumour purity, local immune infiltration, and other site-specific effects.

**Figure 6:**
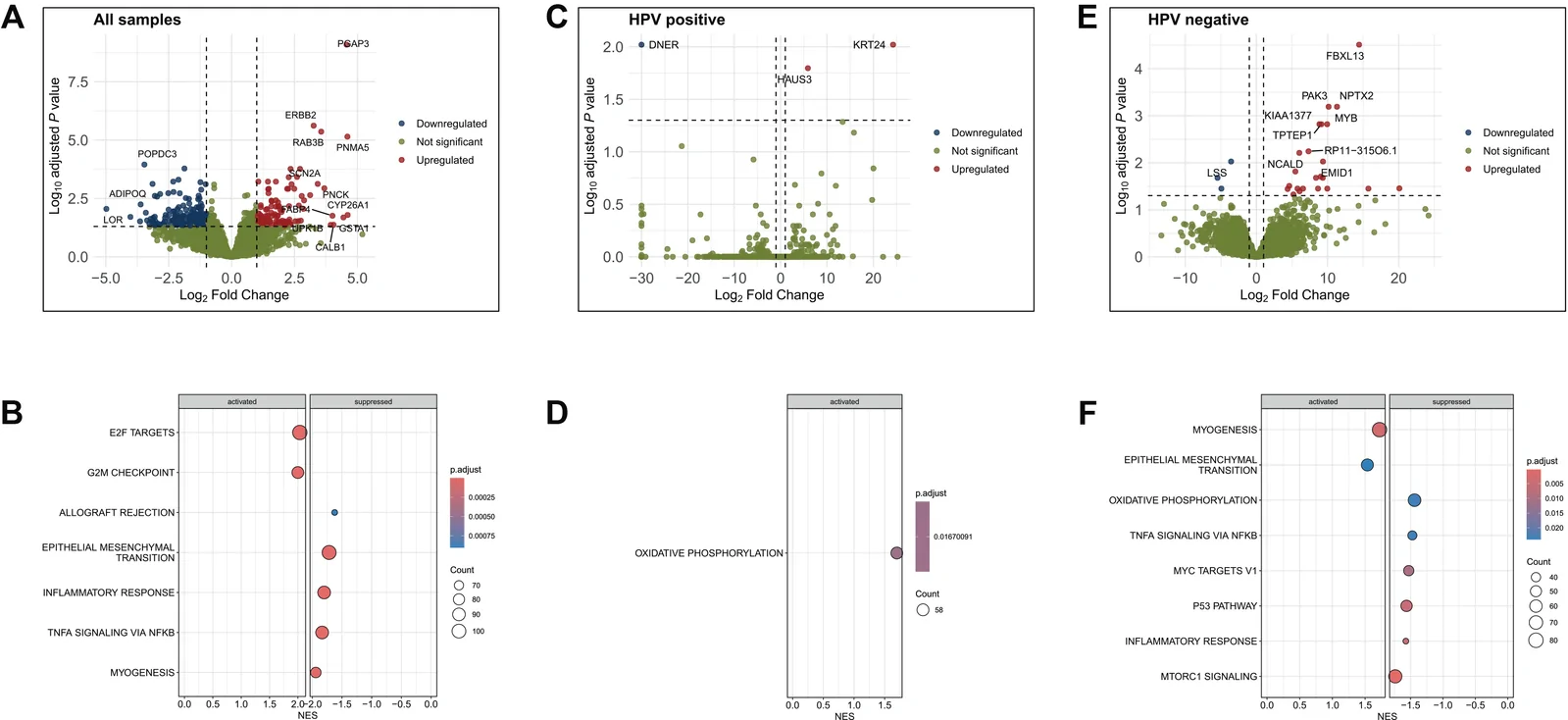
**Gene pathway analysis according to molecular ITH.** (A) Volcano plot showing differential gene expression according to gITH score considered as a continuous variable (DESeq2 analysis). The top 20 genes with the lowest adjusted *P* values are labelled. Thresholds for significance: adjusted *P* < 0.05 and absolute log2 fold change > 1.(B) GSEA using Hallmark gene sets, carried out on DESeq2 differential expression results according to gITH score. Gene sets with false discovery rate-adjusted *P* < 0.05 were considered significantly enriched. (C) Volcano plot showing differential gene expression according to I-TED score considered as a continuous variable (DESeq2 analysis) in patients who are HPV-positive. The top 20 genes with the lowest adjusted *P* values are labelled. Thresholds for significance: adjusted *P* < 0.05 and absolute log2 fold change > 1. (D) GSEA using Hallmark gene sets, carried out on DESeq2^28^ differential expression results according to I-TED score in patients who are HPV-positive. Gene sets with false discovery rate-adjusted *P* < 0.05 were considered significantly enriched. (E) Volcano plot showing differential gene expression according to I-TED score considered as a continuous variable (DESeq2 analysis) in patients who are HPV-negative. The top 20 genes with the lowest adjusted *P* values are labelled. Thresholds for significance: adjusted *P* < 0.05 and absolute log2 fold change > 1. (F) GSEA using Hallmark gene sets, carried out on DESeq2 differential expression results according to I-TED score in patients who are HPV-negative. Gene sets with false discovery rate-adjusted *P* < 0.05 were considered significantly enriched. gITH, genomic ITH; GSEA, gene set enrichment analysis; HPV, human papillomavirus; I-TED, intratumour expression distance; ITH, intratumoral heterogeneity; NES, normalised enrichment score.

We first identified genes whose expression levels were associated with gITH across all tumours (Figure 6A). *ERBB2* expression showed a positive correlation with gITH, as did *PGAP3*, its head-to-head genomic neighbour at 17q21 location. Similarly, *RAB3b*—previously implicated in tumour heterogeneity and aggressiveness^29^—was also associated with increased gITH. Conversely, low expression of Loricrin encoded by *LOR* gene—a marker of differentiated keratinocytes^30^—and *ADIPOQ* whose downregulation has been linked to cancer susceptibility,^31^ was associated with lower gITH. At the pathway level,^32^^,^^33^ increased gITH was linked to heightened cell proliferation and cycling (E2F targets, G2M checkpoint), whereas decreased expression of epithelial–mesenchymal transition (EMT) and inflammatory response-associated genes were observed (Figure 6B).

We then assessed the relationship between gene expression and transcriptomic heterogeneity. In HPV-positive tumours, a few genes were differentially expressed in relation to tITH (Figure 6C), although the oxidative phosphorylation pathway was notably enriched (Figure 6D). In contrast, HPV-negative tumours exhibited a broader set of tITH-associated genes (Figure 6E), including *PAK3*, *NPTX2*, and *MYB*, which have been implicated in tumour aggressiveness and survival.^34^, ^35^, ^36^, ^37^ In these tumours, higher tITH was linked to increased EMT; reduced immune-related signalling; and downregulation of the p53, MYC, and oxidative phosphorylation pathways (Figure 6F).

Taken together, these findings suggest that specific gene-expression patterns and pathway-level alterations are associated with either genomic or transcriptomic ITH.

## Discussion

ITH is increasingly recognised as a major obstacle to effective cancer therapy, underpinning tumour adaptability and acquired resistance mechanisms. In this study, we report, to our knowledge, the largest paired DNA/RNA multiregional sequencing analysis conducted to date in localised HNSCC,^7^^,^^38^^,^^39^ providing a comprehensive view of the spatial genomic and transcriptomic complexity of these tumours. With a median clinical follow-up of 5 years, our dataset also allows assessment of long-term clinical outcomes in relation to spatial molecular features. We assessed the prognostic value of a pathology-based spatial heterogeneity classification, which showed limited predictive power. In contrast, genomic heterogeneity was frequent across tumours, likely reflecting spatial diversification of clonal architecture. However, when accounting for model instability using penalised regression and bootstrap-based internal validation, the association between gITH and DFS was attenuated and was not statistically robust. These findings suggest that, although gITH reflects relevant aspects of tumour complexity, its prognostic effect in this cohort is not confirmed under conservative modelling approaches. tITH was higher in HPV-independent tumours but was not associated with prognosis. Finally, pathways related to cell proliferation, metabolism, and immune response varied significantly with both genomic and tITH, underscoring the complex interplay between molecular diversity and tumour behaviour.

To explore potential genomic drivers of heterogeneity, we assessed associations between gene mutations and clonal diversity. Among the four genes significantly associated with increased gITH, *KMT2c*—a key regulator of gene expression through epigenetic mechanisms—emerged with the strongest signal. Its mutation was associated with a marked increase in heterogeneity. Strikingly, *KMT2c* inactivation has been linked to defects in DNA damage repair and elevated APOBEC mutagenesis across cancer types.^40^, ^41^, ^42^ Moreover, its tumour suppressor role in HNSCC has recently been reinforced^43^ and its mutation has been associated with enhanced response to immune checkpoint blockade.^44^ These observations suggest that *KMT2C* may contribute to shaping tumour clonal architecture and reflect underlying evolutionary processes in HNSCC.

Transcriptomic ITH remains less characterised, particularly in localised HNSCC. We observed a marked difference in tITH between HPV-positive and HPV-negative tumours. In agreement with previous findings, we also found a higher gITH in HPV-negative tumours.^10^ This further supports the notion that HPV-driven oncogenesis follows a distinct evolutionary trajectory, with limited genomic disorder and increased phenotypic stability, which may contribute to the better therapeutic responses observed in this subgroup.

In HPV-positive tumours, both gITH and tITH showed inverse associations with estimated endothelial cell infiltration and tITH correlated with upregulation of oxidative phosphorylation pathway. We also observed elevated haemoglobin gene expression in high-tITH tumours, potentially reflecting a response to hypoxia. Previous studies have linked hypoxic stress and increased ITH^45^^,^^46^ raising the possibility that hypoxia may contribute to tumour diversification in these tumours. However, our analyses relied on bulk RNA-seq deconvolution approaches, which may be influenced by tumour purity and stromal content. Therefore, these observations should be considered exploratory and hypothesis generating. Further spatially resolved or single-cell analyses will be required to clarify the relationship between hypoxia, vascular composition, and tumour heterogeneity in HPV-driven HNSCC. Interestingly, a relationship between HPV dependency and tumour response to bevacizumab is well documented in cervical cancer,^47^ although the relevance of antiangiogenic strategies in HNSCC remains unclear. Overall, our findings suggest that tumour microenvironmental features may contribute to the biological variability associated with HPV-driven tumours.

Several limitations related to heterogeneity quantification should also be acknowledged. Both gITH and I-TED metrics may be influenced by tumour purity, sequencing depth, and the number of sampled tumour regions. In particular, multiregion sampling density may affect the sensitivity for detecting rare genomic subclones or transcriptionally distinct populations. Although all samples were processed using standardised sequencing and analytical pipelines, and I-TED calculations were stratified by HPV status to reduce transcriptional confounding, residual technical variability cannot be excluded. Therefore, these metrics should be interpreted as estimates of intratumour heterogeneity rather than exhaustive representations of tumour complexity.

In addition, tumour-level gITH, defined as the maximum regional Shannon index across sampled regions, captures peak rather than global tumour diversity and may therefore be influenced by spatially restricted outlier regions. More generally, the Shannon index integrates clonal richness and evenness into a single summary measure, which facilitates comparisons across tumours but limits biological interpretability, as distinct evolutionary configurations may yield similar values. Importantly, this approach does not capture the spatial organisation of subclones across tumour regions, which represents an additional dimension of heterogeneity not addressed in the present study.

Alternative tumour-level summary metrics, such as the mean regional Shannon index, Simpson’s diversity index, or other measures of clonal diversity, were not empirically evaluated in the present study. As the raw sequencing data and analysis code are publicly available, future studies can readily assess the robustness of our findings using alternative heterogeneity metrics and analytical approaches.

Overall, our study provides a comprehensive characterisation of spatial genomic and tITH in localised HNSCC. Although genomic heterogeneity reflects substantial biological diversity across tumours, its prognostic impact was not confirmed after conservative statistical correction, highlighting the importance of robust modelling approaches in limited-sample multiregion studies. Our findings nonetheless support the view that tumour evolutionary diversity appears to differ according to HPV status and underscore the need for spatially resolved and functionally validated approaches to fully elucidate the role of intratumour heterogeneity in HNSCC.

## Declaration of AI and AI-Assisted technologies in the writing process

During the preparation of this work, the authors used ChatGPT (OpenAI) for support with code development and writing assistance. After using this tool, the authors reviewed and edited the content as needed and take full responsibility for the content of the published article.

## Supplementary data

Supplementary Figures S1-S5

Detailed Methods
