# Supplementary material for: Multiregion profiling of genomic and transcriptional heterogeneity in head and neck squamous-cell carcinoma
Source: ESMO Open. 2026 Sep 11;11(10):108367. doi: 10.1016/j.esmoop.2026.108367 (PMC13583575; doi:10.1016/j.esmoop.2026.108367)
Supplement: Supplementary file 1 [file mmc3.pdf]

ID-RCB 2016-A01095-46

IC 2016-03

**Prospective biobanking study in cancer patients aiming at better understand the link between the molecular alterations of the tumor itself, its microenvironment and immune response**

Protocol running title:

"SCANDARE"

Version n° 1.0 – June 24th, 2016 – (sent to EC and ANSM)
Version n° 1.0 – June 24th, 2016 – (Approved by EC and ANSM)
Version n°2.0 – July 6th, 2018 – (sent to EC and ANSM)
Version n° 2.0 – July 6th, 2018 – (Approved by EC and ANSM)
Version n°3.0 – June 4th, 2019 – (not sent to EC and ANSM)
Version n°4.0 – July 9th, 2019 – (not sent to EC and ANSM)
Version n°5.0 – September 10th, 2020 – (sent to EC and ANSM)
Version n°5.0 – September 10th, 2020 – (Approved by EC and ANSM)
Version n°6.0 – October 21th, 2021 – (Sent to EC and ANSM)
Version n°6.0 – October 21th, 2021 – (Approved by EC and ANSM)
Version 7.0 – February 15th, 2023 – (Sent to EC and ANSM)
Version 7.0 – February 15th, 2023 – (Approved by EC and ANSM)

Version 8.0 – February 25th 2025 (Sent to EC and ANSM)
Version 8.0 – February 25th 2025 (Approved by EC and ANSM)

**INVESTIGATOR COORDINATOR**	**Pr Christophe Le Tourneau**<br>Institut Curie – 26 rue d'Ulm – 75005 Paris<br>Tél. : 0144324086<br>Fax : 0153104006<br>Email : christophe.letourneau@curie.fr

**SPONSOR**	**INSTITUT CURIE**<br>26, rue d'Ulm – 75248 PARIS Cedex 05

APPROVAL AND RESPONSIBILITIES

Title: Prospective biobanking study in cancer patients aiming at better understand the link between the molecular alterations of the tumor itself, its microenvironment and immune response

**COMPETENT AUTHORITY**	**National Agency for the Safety of Drugs and Health Products (ANSM)**	**Date of authorization: July 18th 2016**
		**Ref. ANSM: ID-RCB 2016-A01095-46**
**ETHICS COMMITTEE**	**Name of the committee for the protection of Persons (CPP): CCP Ile de France III**	**Date of approval: December 9th 2016**
		**Ref. CPP : n°3440**

**SCIENTIFIC COORDINATOR**	Constance Lamy, PhD
**WRITING COMMITTEE**	Christophe Le Tourneau, Emanuela Romano, Maud Kamal, Ivan Bièche, Gaëlle Pierron, Olivier Delattre, Philippe Hupé, Nicolas Servant, Jean-Yves Pierga, François-Clément Bidard, Anne-Vincent Salomon, Sébastien Armanet, Anne-Sophie Plissonnier, Vassili Soumelis, Olivier Lantz, Delphine Loirat, Sergio Roman Roman, Roman Rouzier, Emmanuel Barillot, Fatima Mechta-Grigoriou, Patricia Tresca, François Doz, Gudrun Schleiermacher, Céline Callens, Marie-Paule Sablin, Marie Alt ; Amir Kadi, Frédérique Berger, Linda Larbi Cherif, Constance Lamy.

NAMES AND RESPONSABILITIES	**CONTACTS**	**DATE (JJ-MM-AA)**	**SIGNATURE**
**Legal representative of the sponsor**	Pr. Steven LE GOUILL, MD PhD<br>Et par délégation,<br>Pr. Xavier PAOLETTI, PhD	27 février 2025	DocuSigned by: Pr. Xavier Paoletti
**Coordinator**	Pr Christophe LE TOURNEAU, MD, PhD	27 février 2025	DocuSigned by: Prof. Christophe Le Tourneau
**Methodologist**	Frédérique BERGER	27 février 2025	DocuSigned by: Frédérique BERGER

SYNOPSIS – PROTOCOL N° IC – 2016-03

ID-RCB 2016-A01095-46

A) CLINICAL TRIAL IDENTIFICATION	
**Protocol number:** IC 2016-03<br>**Version and date:** Version 8.0 – 25/02/2025	
**Title of the trial:** Prospective biobanking study in cancer patients aiming at better understand the link between the molecular alterations of the tumor itself, its microenvironment and immune response	
**Running title:** SCANDARE	
**Coordinator:** Christophe Le Tourneau	
**Estimated number of centers in France:** 4-6	**Number of patients:** 1050

B) SPONSOR IDENTIFICATION
INSTITUT CURIE<br>26, rue d'Ulm – 75248 PARIS Cedex 05
Pr Steven LE GOUILL,<br>Director of the Hospital Group<br>**Phone:**+33-1 56 24 56 65– **Fax:**+33-1 53 10 40 29 – **Email:** drci.promotion@curie.fr

C) GENERAL TRIAL INFORMATION
**Indication:** Early stage treatment-naive ovarian cancer, triple-negative breast, head and neck cancers, advanced stage treatment-naïve cervical cancer and sarcoma (breast angiosarcoma and uterine sarcoma) cancers.
**Methodology:** Prospective biobanking study on tumor, plasma and blood samples at different time points
**Primary objective of the study:** To better understand the link between the molecular alterations of the tumor itself, its microenvironment and immune response.
**Secondary objectives:**<br>➢ To characterize the composition of the tumor microenvironment (TME) and cellular communication before and after treatment<br>➢ To assess the links between the tumor molecular profile, the TME and the immune cells<br>➢ To analyze the impact of TME parameters on sensitivity/response to treatment<br>➢ To correlate temporal and spatial tumor heterogeneity with immune activation<br>➢ To assess the predictive value of circulating biomarkers on sensitivity or resistance to

treatment

- For cervical cancer patients : to identify and validate new molecular and/or immunological biomarkers associated with clinical and biological features of the tumors

Inclusion criteria:

1) Tumor types:
    a. Newly diagnosed treatment-naïve ovarian cancer patients eligible for surgery or neoadjuvant chemotherapy
    b. Newly diagnosed treatment-naïve triple-negative breast cancer patients eligible for surgery or neoadjuvant chemotherapy
    c. Newly diagnosed treatment-naïve head and neck cancer patients eligible for surgery
    d. Newly diagnosed treatment-naïve cervical cancer patients (1) stage Ia – IIa1 with nodal metastasis, postoperative positive margin or parametrial-vaginal involvement, and 2) stage ≥IIa2).
    e. Newly diagnosed treatment-naïve sarcoma cancer patients (1) breast angiosarcoma or (2) uterine sarcoma eligible for surgery or systemic treatment
2) Male or female patients ≥ 18 years of age
3) Signed informed consent

Exclusion criteria:

1) Male or female patients ≤18 years old
2) Patients with any psychological, familial, sociological or geographical condition potentially hampering compliance with the study protocol and follow-up schedule
3) Individually deprived of liberty or placed under the authority of a tutor
4) Patients not affiliated to the Social Security System

Principal Evaluation Criteria:

Association between molecular and/or immunological biomarkers and baseline clinicobiological features of the tumors

Secondary Evaluation Criteria:

- Recurrence at 2 years associated with molecular and/or immunological biomarkers
- Correlation between genomic alterations and immune parameters
- Correlation between mutations load and immune parameters
- Correlation between ctDNA levels, de novo mutations in ctDNA and immune parameters
- For cervical cancer patient, correlation between ctDNA levels and kinetics, and prognosis, prediction of recurrence

D) STUDY DESIGN
All patients will receive standard treatment according to the stage of the diseases and usual procedures.

E) STATISTICAL and BIOINFORMATICS ANALYSES

Sample Size: This is an exploratory study; no hypothesis could be made regarding the number of patients needed to achieve any of the study's objectives. Based on the feasibility of the sampling, on financial and logistical constraints and on enrollment possibilities, a total of 500 patients with triple negative breast cancer or ovarian cancer and 300 patients with head and neck cancer are required. Moreover, based on previous data from the BioRAIDs (NCT02428842) study, 200 patients with cervical cancer are required also in order to establish the correlations between the different immune or molecular parameters and clinicobiological features and validate previously published prognostic biomarkers. At least, 50 patients with breast angiosarcoma or uterine sarcoma will be enrolled to collect molecular and immunological information associated with the clinical features of these rare diseases.

Two-hundred patients will allow us to highlight the presence of a biomarker in the population with a precision level varying from 3.1% for a rare biomarker (present in 5% of the population) to 6.9% for a biomarker present in 50 % of the population.
With 200 patients, the power of association test performed by Chi-square test will reach between 85% and 99% depending on the observed effect size; the power to observe a medium effect size will be 85 % while it will reach 99% to highlight a large effect size

As angiosarcoma or uterine sarcoma are rare cancers, the limited number of patients with angiosarcoma or uterine sarcoma enrolled is based on the active file of patients diagnosed each year at the Institut Curie. Statistical analyses will be purely exploratory with no pre-specified power.

Criteria of statistical analysis:
No statistical hypothesis applies but the following methods will be used to analyze the results.

- Multiparametric description of TME in human primary TNBC, HNSCC and ovarian tumors
- Statistical analysis of co-segregation of TME parameters and association to clinical features
- Statistical and mechanistic models of cell communication networks within the TME
- Statistical analysis of tumor molecular profile parameters associated to TME characteristics
- Univariate and multivariate statistical analysis of TME parameters associated to response to standard treatment.

Molecular & Bioinformatics analysis:

- DNA sequencing and methylation assessments on baseline tumors and corresponding blood to assess: 1) tumor mutational load, 2) most frequently altered genes and copy number alterations, 3) methylation signatures and 4) patterns of heterogeneity
- High throughput RNA sequencing for expression profiles to study gene expression in tumors and TME.
- Shallow Whole Genome Sequencing (sWGS) to assess copy number (CN) signatures
- Luminex and FACS analyses
- Integrative analyses

F) ESTIMATED TRIAL DURATION

Inclusion period: 11 years

Follow up period: 3 years for ovarian, head and neck and cervical cancer patients
5 years for triple negative breast and sarcoma cancer patients

Total trial duration: 16 years maximum

LIST OF ABBREVIATIONS

CPP : Committee for the Protection of Persons
ctDNA : circulating tumor DeoxyriboNucleic Acid
DC : Dentritic Cell
eCRF : electronic Case Report Form
EDTA : Ethylene Diamine Tetraacetic Acid
FACS : Fluorescence Activated Cell Sorting
FFPE : Formalin-Fixed Paraffin-Embedded
GCP : good clinical practice
HNSCC : Head and Neck Squamous Cell Carcinoma
MTAs : Molecularly Targeted Agents
NGS : Next-Generation Sequencing
NK : Natural Killer
PM : Personalized Medicine
RNA : RiboNucleic Acid
TME : Tumor Microenvironement
TNBC : Triple Negative Breast Cancer

TABLE OF CONTENTS

1. INTRODUCTION AND RATIONALE ... 10
1.1 Introduction ... 10
1.2 Rationale ... 10
2. STUDY OBJECTIVES AND ENDPOINTS ... 11
2.1 Primary objective ... 11
2.2 Secondary objectives ... 11
2.3 Principal Evaluation Criteria: ... 11
2.4 Secondary Evaluation Criteria: ... 11
3. PATIENT SELECTION ... 12
3.1 Patient selection ... 12
3.2 Patients' registration ... 13
3.3 Patients' evaluation ... 13
3.3.1 For newly diagnosed treatment-naïve ovarian cancer patients, triple negative breast cancer patients, Head and neck cancer patients upfront surgery ... 13
3.3.2 For newly diagnosed treatment-naïve cervical cancer patients (1) stage Ia – IIa1 with nodal metastasis, postoperative positive margin or parametrial-vaginal involvement, and 2) stage ≥IIa2 ... 14
3.3.3 For newly diagnosed treatment-naïve ovarian cancer patients, triple negative breast cancer patients before neoadjuvant chemotherapy ... 15
3.3.4 For newly diagnosed treatment-naïve sarcoma cancer patients, before surgery ... 16
3.3.5 For newly diagnosed treatment-naïve sarcoma cancer patients, before systemic treatment 16
3.4 Length of study / end of study ... 17
4. SAMPLE COLLECTION ... 17
4.1 Tissue Sampling Schedule ... 17
4.1.1 Tumor and lymph node samples: ... 17
4.1.1.1 For newly diagnosed treatment-naïve ovarian cancer, triple negative breast cancer and head and neck cancer patients upfront surgery ... 17
4.1.1.2 For newly diagnosed treatment-naïve cervical cancer patients ... 17
4.1.1.3 For newly diagnosed treatment-naïve ovarian cancer patients, before neoadjuvant chemotherapy ... 18
4.1.1.4 For newly diagnosed treatment-naïve triple negative breast cancer patients, before neoadjuvant chemotherapy ... 18
4.1.1.5 For newly diagnosed treatment-naïve sarcoma cancer patients upfront surgery ... 19
4.1.1.6 For newly diagnosed treatment-naïve sarcoma cancer patients before systemic treatment 19
4.1.1.7 Tumor and lymph nodes sampling conditions: ... 19
4.1.2 Blood sampling ... 21
4.1.2.1 For newly diagnosed treatment-naïve ovarian cancer patients, triple negative breast cancer patients and Head and neck cancer patients upfront surgery ... 21
***4.1.2.2 For newly diagnosed treatment-naïve cervical cancer patients** ... 22*
4.1.2.3 For newly diagnosed treatment-naïve ovarian cancer patients, triple negative breast cancer patients before neoadjuvant chemotherapy ... 22
***4.1.2.4 For newly diagnosed treatment-naïve sarcoma cancer patients upfront surgery** ... 23*
4.1.2.5 For newly diagnosed treatment-naïve sarcoma cancer patients before systemic treatment 23
4.2 Tissue Sampling Management ... 24
5. MOLECULAR, TUMOR MICROENVIRONMENT AND IMMUNE ANALYSES ... 24
5.1 Molecular analysis on the tumor biopsies ... 24
5.2 Molecular analysis on circulating tumor DNA (ctDNA) ... 24
5.3 Tumor MicroEnvironment (TME) analysis ... 24
5.4 Immune analysis ... 25
6. STUDY DESIGN ... 25
7. RECOMMENDATIONS FOR INDIVIDUAL TREATMENT OPTIONS ... 25

8. SAFETY ASSESSEMENT 26
8.1 General Definition 26
8.2 Intensity Criteria 26
8.3 Reporting Serious Adverse Event 27
8.4 Serious Adverse Event Follow-up 27
9. STATISTICAL CONSIDERATIONS 27
9.1 Sample size and power considerations 27
9.2 Statistical Analysis 28
10. DATA PROTECTION AND CONFIDENTIALITY MANAGEMENT 29
11. QUALITY ASSURANCE AND QUALITY CONTROL 29
11.1 Data Safety Monitoring Board (DSMB) 29
11.2 Quality control 29
12. ETHICAL AND REGULATORY ISSUES 29
12.1 Ethics Committee 30
12.2 Competent authority 30
12.3 Information and consent of the participants 30
12.4 Responsibilities of the sponsor 31
12.5 Responsibilities of the investigators 31
12.6 Federation of the Patient Committees for Clinical Research in Cancerology 32
13. PUBLICATION OF RESULTS 32
14. FUNDING AND INSURANCE 33
14.1 Funding 33
14.2 Insurance 33
15. REFERENCES 33
16. APPENDIX 34
APPENDIX 1 : PATIENTS SAMPLES 34
APPENDIX 2 : Techniques used per sample 36
APPENDIX 3 - Toxicity criteria (CTCAE) 37

1. INTRODUCTION AND RATIONALE

1.1 Introduction

The last decade witnessed the development of high-throughput technologies such as microarrays and next-generation sequencing (NGS) which paved the way to precision medicine in the field of oncology. Precision medicine also referred to as personalized medicine (PM) is defined by the National Cancer Institute as “A form of medicine that uses information about a person’s genes, proteins, and environment to prevent, diagnose, and treat disease”. In the field of oncology, PM emerged 20 years ago with the development of molecularly targeted agents (MTAs) such as tyrosine-kinase inhibitors and monoclonal antibodies. Centers for personalized and PM have been created in several comprehensive cancer centers over the world.

Nevertheless, several reasons hindered the massive deployment of MTAs towards histology-agnostic cancer treatment in clinical practice among which the lack of validated biomarkers and a clear functional relevance for a specific molecular alteration identified in a tumor usually harboring several other molecular alterations.

Several questions remain to be answered in the context of PM, including the challenges of tumor heterogeneity and the emergence of resistance mechanisms as well as the complexity and influence of the tumor microenvironment (TME), the immune mechanisms or the epigenome.

1.2 Rationale

The concept that tumor-host interactions are crucial in tumor progression is now well accepted. It has become increasingly clear in many cancer types that tumors are complex ecologies that are affected by numerous stromal factors that dampen or enhance the effects of genomic epithelial alterations. Recent studies indicate that TME and immune response can help in determining clinical outcome in human cancer patients. Indeed, TME may also confer drug-resistance and treatment failure, leading to tumor recurrence and metastasis. Some TME components (including cancer associated fibroblasts and lymphocytes) have been shown to contribute to sensitivity and/or resistance to conventional chemotherapies and targeted therapeutic agents but further investigations are required to delineate TME contribution to treatment sensitivity or resistance.[1, 2, 3, 4]

However, to date, PM has been mostly focused on the assessment of genomic variants to facilitate selection of drug combinations likely to benefit specific patients. Proof of concept of the value of assessing immune parameters in predicting response to therapy has been obtained in checkpoint blocker clinical studies. The impressive clinical activity of immune-checkpoint-pathway inhibitors such as anti–CTLA-4 (ipilimumab) [5] and anti–programmed death 1 (PD-1) [6, 7] monoclonal antibody in a variety of solid tumors emphasizing the fact that not all actionable cancer targets are genomic alterations.

In summary, these molecules have important characteristics, which differentiate them from some other therapies in oncology:
1) They act in many types of tumors from different anatomical location. This implies a paradigm shift as compared to other treatment strategies dictated by tumor type.
2) They induce significant proportions of partial and complete responses (20-40%) across different and independent clinical trials. This is a major difference from other immunotherapy strategies.

3) They induce long-term responses and disease stabilization, with relapse free survival of over two years in some metastatic patients.
Despite the impressive clinical results, checkpoint inhibitors and immunotherapy at large benefits on a small fraction of treated patients. Hence, the urgent need of combinatorial, longitudinal approaches that allow multiparameter immune monitoring of tumors and blood to capture the complex therapeutic effects of therapy on host immunity and the TME. This together with data from NGS will provide a road-map for the design of combinatorial approaches for the treatment of cancer.

SCANDARE is an Institut Curie initiative and collaboration between several teams of the Hospital and the Research center focusing on the tumor, its microenvironment and the immune parameters in selected tumor types. The choice of breast, ovarian, head and neck, cervical and sarcoma (angiosarcoma or uterine sarcoma) cancers for the SCANDARE study are based on the accessibility of these types of cancer at Institut Curie as well as the scientific expertise of the different teams involved in this project.

SCANDARE aims to better understand the link between the molecular alterations of the tumor itself and its microenvironment via the set-up of a “precision medicine” prospective biobanking study.

2. STUDY OBJECTIVES AND ENDPOINTS

2.1 Primary objective

To better understand the link between the molecular alterations of the tumor itself, its microenvironment and immune response.

2.2 Secondary objectives

- To characterize the composition of the tumor microenvironment (TME) and cellular communication before and after treatment
- To assess the links between the tumor molecular profile, the TME and the immune cells
- To analyze the impact of TME parameters on sensitivity/response to treatment
- To correlate temporal and spatial tumor heterogeneity with immune activation
- To assess the predictive value of circulating biomarkers on sensitivity or resistance to treatment
- To identify and validate new molecular and/or immunological biomarkers associated with clinical and biological features of the tumors

2.3 Principal Evaluation Criteria:

Association between molecular and/or immunological biomarkers and baseline clinicobiological features of the tumors

2.4 Secondary Evaluation Criteria:

- Recurrence at 2 years associated with molecular and/or immunological biomarkers
- Correlation between genomic alterations and immune parameters

- Correlation between mutations load and immune parameters
- Correlation between ctDNA levels, de novo mutations in ctDNA and immune parameters
- Correlation between ctDNA levels and kinetics, and prognosis, prediction of recurrence

3. PATIENT SELECTION

3.1 Patient selection

The patients must fulfill the following inclusion and exclusion criteria:

Inclusion criteria:

1) Tumor types :
    a. Newly diagnosed treatment-naïve ovarian cancer patients eligible for surgery or neoadjuvant chemotherapy
    b. Newly diagnosed treatment-naïve triple-negative breast cancer patients eligible for surgery or neoadjuvant chemotherapy
    c. Newly diagnosed treatment-naïve head and neck cancer patients eligible for surgery
    d. Newly diagnosed treatment-naïve cervical cancer patients (1) stage Ia – IIa1 with nodal metastasis, postoperative positive margin or parametrial-vaginal involvement, and 2) stage ≥IIa2
    e. Newly diagnosed treatment-naïve sarcoma cancer patients (1) breast angiosarcoma or (2) uterine sarcoma eligible for surgery or systemic treatment

2) Male or female patients ≥ 18 years of age
3) Signed informed consent

Exclusion criteria:

1) Male or female patients ≤18 years old
2) Patients with any psychological, familial, sociological or geographical condition potentially hampering compliance with the study protocol and follow-up schedule
3) Individually deprived of liberty or placed under the authority of a tutor
4) Patients not affiliated to the Social Security System

3.2 Patients' registration

Following the signature of an informed consent and the validation of the inclusion assessments, eligible patients will be registered in the eCRF on the day of tissue sampling at the following address:

https://ecrf.curie.net

The access password shall be delivered to each center at the beginning of the Study.

3.3 Patients' evaluation

Following clinical data will be recorded:

In case of neoadjuvant chemotherapy, exclusive (chemo) radiotherapy or chemotherapy: clinical, radiological and pathological response to therapy

In case of primary surgery: disease-free survival data as per clinical routine

In all cases: overall survival data

3.3.1 For newly diagnosed treatment-naïve ovarian cancer patients, triple negative breast cancer patients, Head and neck cancer patients upfront surgery

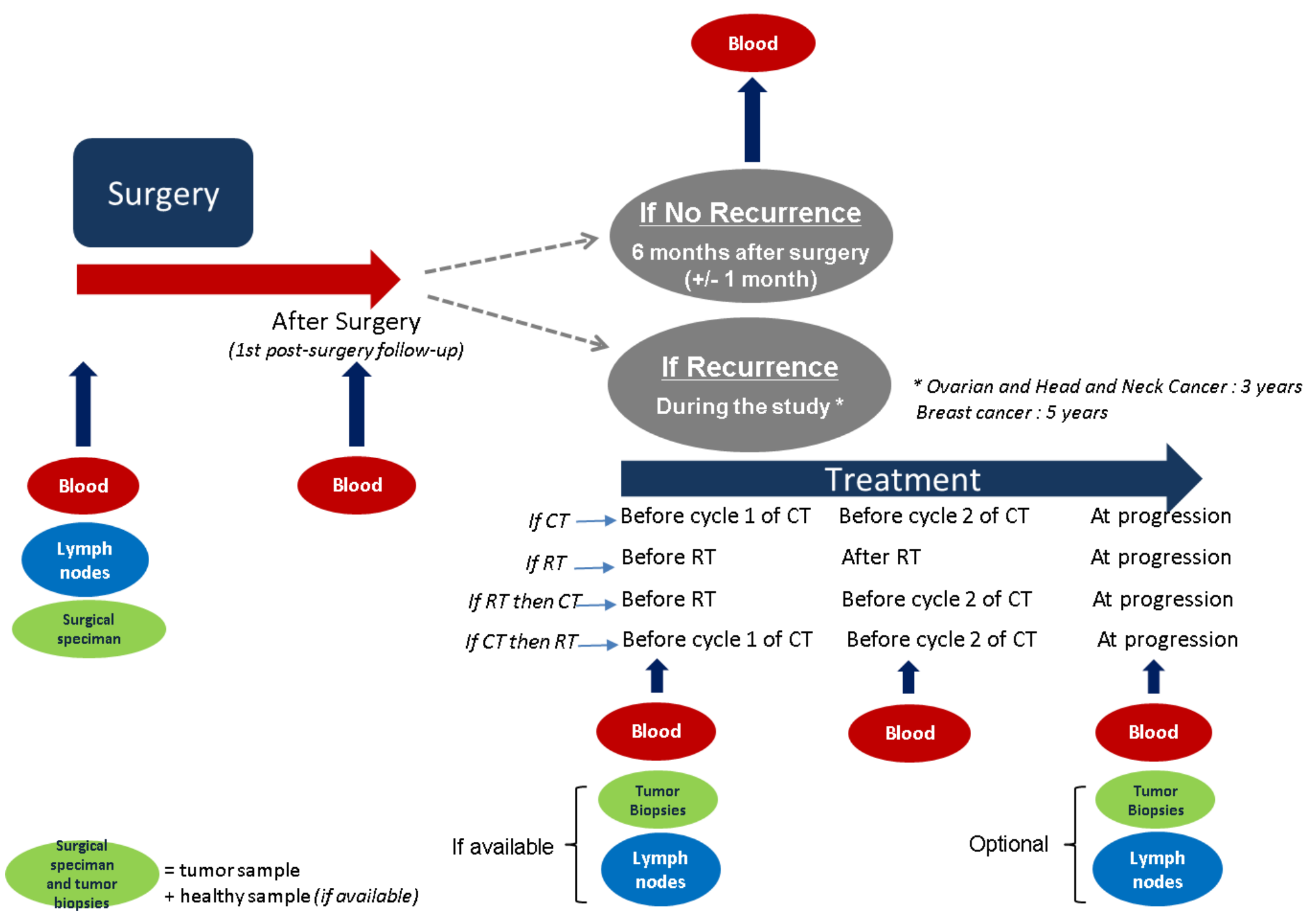

3.3.2 For newly diagnosed treatment-naïve cervical cancer patients (1) stage Ia – IIa1 with nodal metastasis, postoperative positive margin or parametrial-vaginal involvement, and 2) stage ≥IIa2

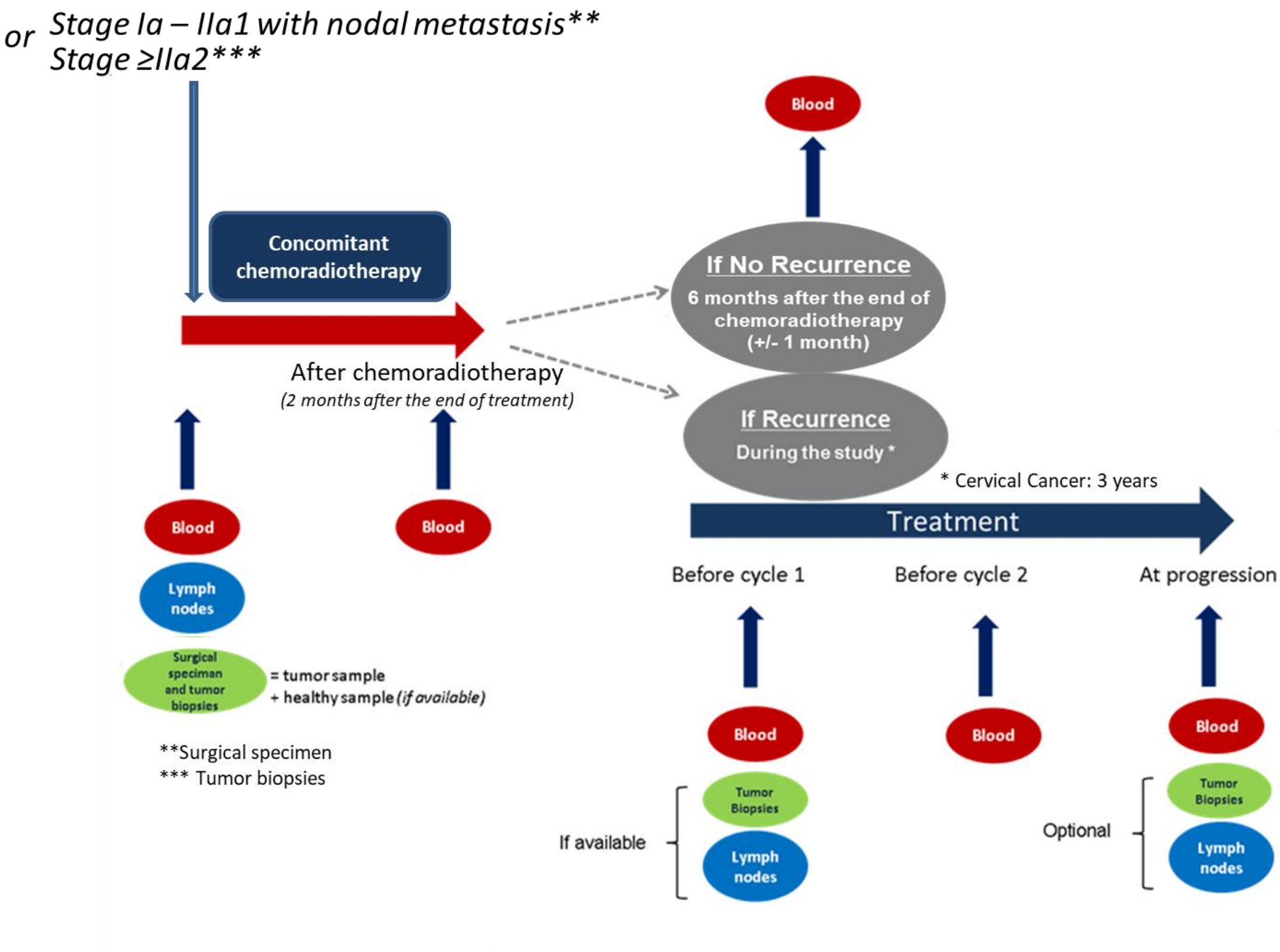

3.3.3 For newly diagnosed treatment-naïve ovarian cancer patients, triple negative breast cancer patients before neoadjuvant chemotherapy

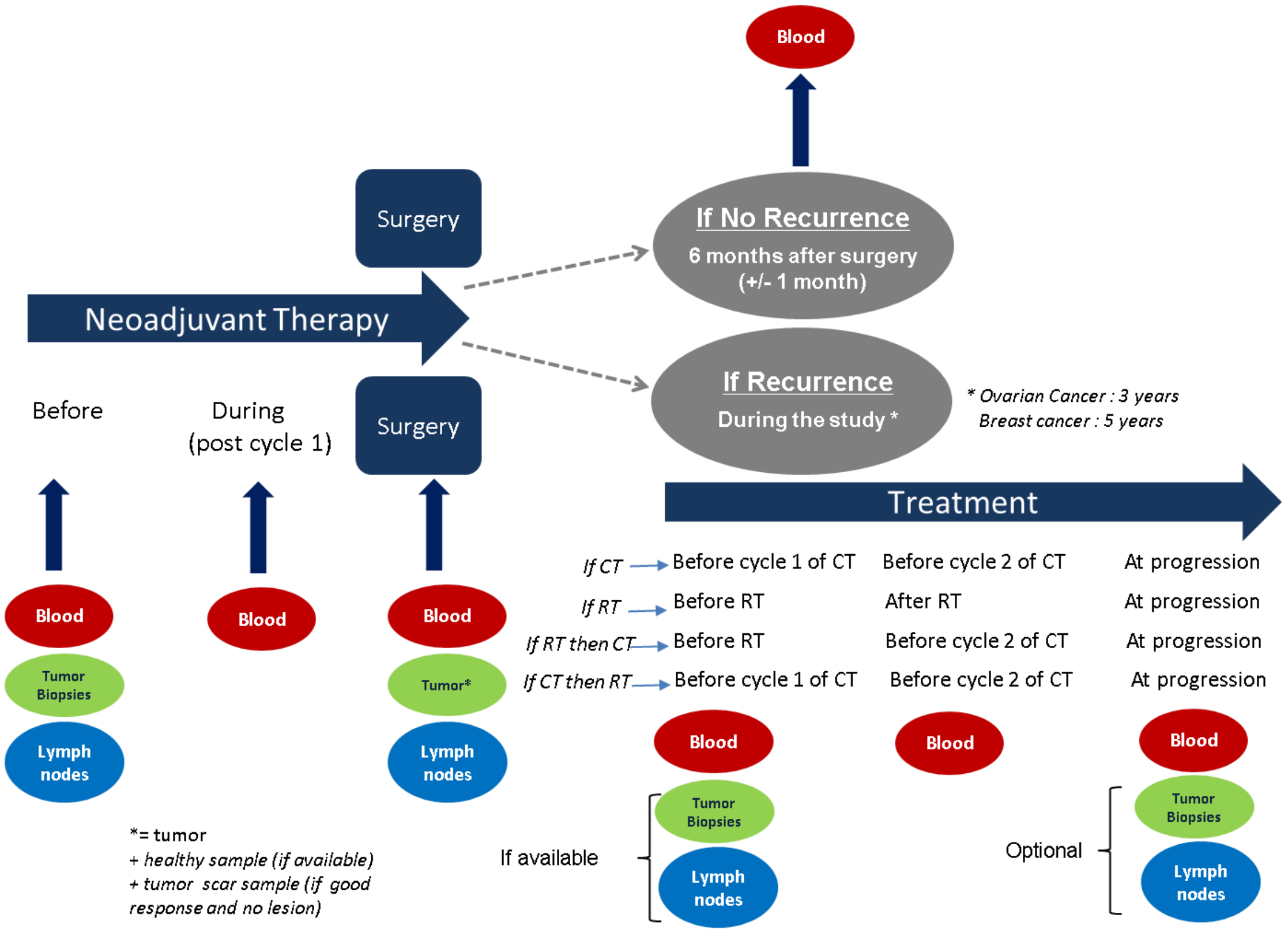

3.3.4 For newly diagnosed treatment-naïve sarcoma cancer patients, before surgery

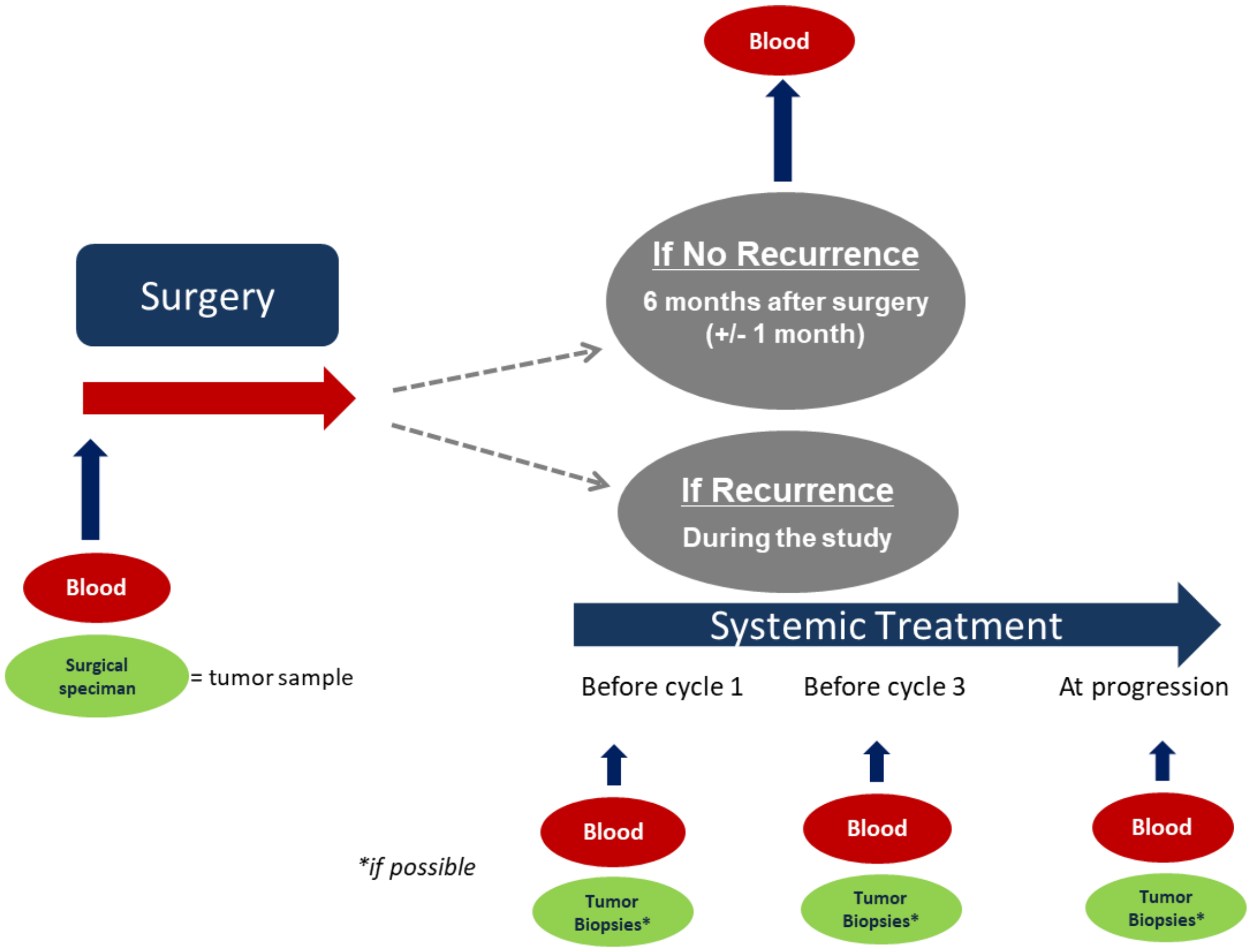

3.3.5 For newly diagnosed treatment-naïve sarcoma cancer patients, before systemic treatment

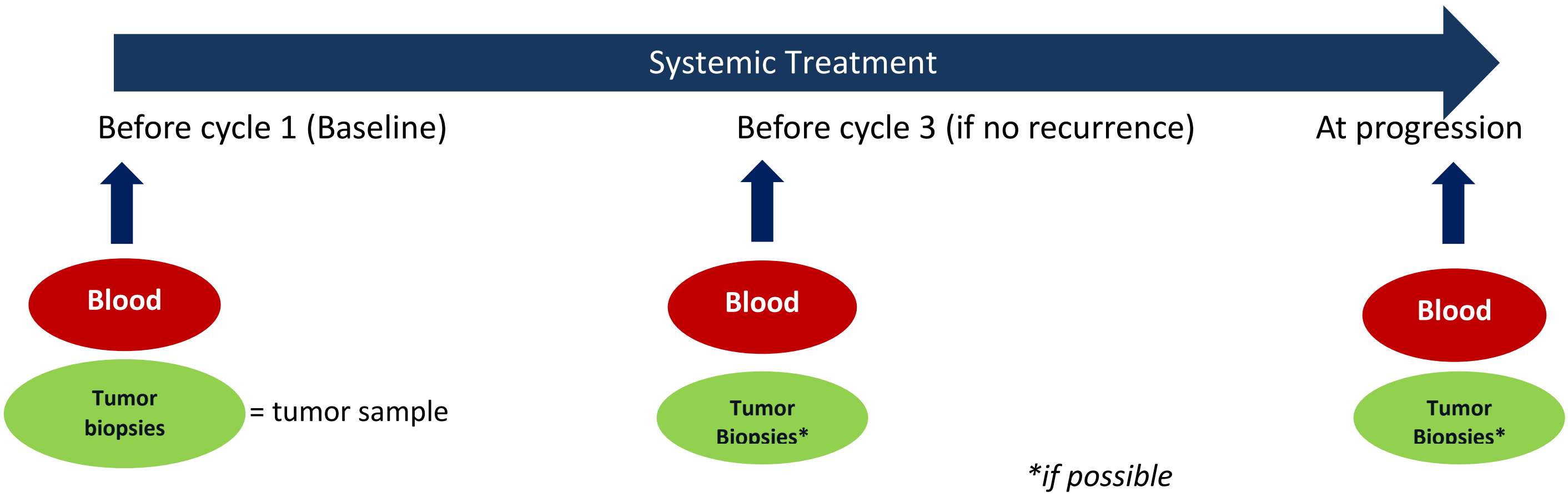

3.4 Length of study / end of study

This study will last 16 years with an inclusion period of 11 years.

At the individual levels, ovarian, head and neck and cervical cancer patients will be followed up during 3 years and triple negative breast and sarcoma cancer (angiosarcoma or uterine sarcoma) patients will be followed up during 5 years.

The participation to the study can be discontinued at any time, following a decision of either the patient or investigator.

4. SAMPLE COLLECTION

Please refer to table 1 in appendix 1

4.1 Tissue Sampling Schedule

For this research study, tissue samples will be collected in all patients included at different time points as indicated in sections 3.3.1 and 3.3.2

Specifically:

4.1.1 Tumor and lymph node samples:

4.1.1.1 For newly diagnosed treatment-naïve ovarian cancer, triple negative breast cancer and head and neck cancer patients upfront surgery

One tumor and lymph node samples composed of at least 4 tumor samples must be collected at the time of primary surgery (be it in a curative intent or not) at the time of recurrence before the start of treatment and at progression if possible:

→ 2 tumor sample and its surrounding normal tissue (if possible) will be paraffin-embedded (FFPE) for Immunohistochemistry analyses

→ 2 tumor sample and its surrounding normal tissue (if possible) will be instantly frozen in liquid nitrogen and stored at -80°C for retrospective Exome and RNA sequencing

→ 1 fresh tumor samples and its surrounding normal tissue (if possible) will be used for immediate TME analyses

4.1.1.2 For newly diagnosed treatment-naïve cervical cancer patients

For stage Ia – IIa1 with nodal metastasis population, one surgical specimen and lymph node samples composed of at least 4 tumor samples must be collected at the time of primary surgery (be it in a curative intent or not) at the time of recurrence before the start of treatment and at progression if possible:

→ 2 tumor sample and its surrounding normal tissue (if possible) will be paraffin-embedded (FFPE) for Immunohistochemistry analyses

→ 2 tumor sample and its surrounding normal tissue (if possible) will be instantly frozen in liquid nitrogen and stored at -80°C for retrospective Exome and RNA sequencing

For stage ≥ IIa population, one tumor biopsie and lymph node samples composed of at least 4 tumor samples must be collected at the time of primary surgery (be it in a curative intent or not) at the time of recurrence before the start of treatment and at progression if possible:

→ 2 tumor sample and its surrounding normal tissue (if possible) will be paraffin-embedded (FFPE) for Immunohistochemistry analyses

→ 2 tumor sample and its surrounding normal tissue (if possible) will be instantly frozen in liquid nitrogen and stored at -80°C for retrospective Exome and RNA sequencing

4.1.1.3 For newly diagnosed treatment-naïve ovarian cancer patients, before neoadjuvant chemotherapy

One tumor and lymph node samples composed of at least 4 tumor samples must be collected at the time of primary surgery (be it in a curative intent or not) at the time of recurrence before the start of treatment and at progression if possible:

→ 2 tumor sample and its surrounding normal tissue will be paraffin-embedded (FFPE) for Immunohistochemistry analyses

→ 2 tumor sample and its surrounding normal tissue will be instantly frozen in liquid nitrogen and stored at -80°C for retrospective Exome and RNA sequencing

→ 1 fresh tumor samples and its surrounding normal tissue will be used for immediate TME analyses

If possible, tumor scar tissue will be realized at the same time of tumor sampling at surgery post neoadjuvant chemotherapy in case of good response and no lesion and as stated above.

4.1.1.4 For newly diagnosed treatment-naïve triple negative breast cancer patients, before neoadjuvant chemotherapy

One tumor and lymph node samples composed of at least 4 tumor samples must be collected at the time of biopsy, at the time of surgery post neoadjuvant chemotherapy (be it in a curative intent or not) at the time of recurrence before the start of treatment and at progression if possible:

- At the time of biopsy:

→ 2 tumor sample will be paraffin-embedded (FFPE) for Immunohistochemistry analyses

→ 2 tumor samples will be instantly frozen in liquid nitrogen and stored at -80°C for retrospective Exome and RNA sequencing

→ 1 fresh tumor sample will be used for immediate TME analyses

- At the time of surgery post neoadjuvant chemotherapy, at the time of recurrence before the start of treatment and progression:

→ 2 tumor sample and its surrounding normal tissue will be paraffin-embedded (FFPE) for Immunohistochemistry analyses

→ 2 tumor sample and its surrounding normal tissue will be instantly frozen in liquid nitrogen and stored at -80°C for retrospective Exome and RNA sequencing

→ 1 fresh tumor samples and its surrounding normal tissue will be used for immediate TME analyses

If possible, tumor scar tissue will be realized at the same time of tumor sampling at surgery post neoadjuvant chemotherapy in case of good response and no lesion and as stated above.

4.1.1.5 For newly diagnosed treatment-naïve sarcoma cancer patients upfront surgery

One tumor sample composed of at least 5 tumor samples must be collected at the time of primary surgery (be it in a curative intent or not) at the time of recurrence before the start of treatment, during treatment and at progression if possible:

→ 2 tumor samples (if possible) will be paraffin-embedded (FFPE) for Immunohistochemistry analyses

→ 1 tumor sample (if possible) will be instantly frozen in liquid nitrogen and stored at -80°C for retrospective Exome and RNA sequencing

→ 2 fresh tumor samples (if possible) will be used for immediate TME analyses

4.1.1.6 For newly diagnosed treatment-naïve sarcoma cancer patients before systemic treatment

One tumor sample composed of at least 5 tumor samples must be collected at the time of biopsy before the start of treatment, during treatment before cycle 3 (if no recurrence) and at progression if possible:

→ 2 tumor samples (if possible) will be paraffin-embedded (FFPE) for Immunohistochemistry analyses

→ 1 tumor sample (if possible) will be instantly frozen in liquid nitrogen and stored at -80°C for retrospective Exome and RNA sequencing

→ 2 fresh tumor samples (if possible) will be used for immediate TME analyses

4.1.1.7 Tumor and lymph nodes sampling conditions:

In the SCANDARE study, biopsies will be performed by professionals as part of the routine patient care.

- **Tumor sampling realized during a diagnostic or therapeutic act should be preferred.**
- If not possible, specific tumor sampling for the SCANDARE study can be performed.
- Normal tissue sampling realized at the same time of tumor sampling. There is no additional sample required; the surrounding normal tissue will be collected.

Lymph nodes collection will always be performed in the context of the routine patient care i.e when lymph nodes are removed for diagnostic or therapeutic purposes (e.g. axillary dissection during breast surgery…).
Tumor and lymph node samples should always be used for diagnostic purposes before any research's ones.

If local investigator considers there is not enough tumor material that could be taken for the study, tumor and lymph nodes samples will be collected as followings:

- Priority: Frozen tissue and depending on the size of the biopsy a fresh sample will be taken. In case of surgical specimen the size is usually sufficient for the collection of fresh and frozen material.
- Secondarily: FFPE

In case of multiple site disease (for tumor sampling at disease progression), investigator decides which site should be selected for tumor biopsies according to the following rules:

• When a biopsy is necessary to confirm the diagnosis of metastatic disease (eg: one single metastatic site, no radiological evidence), a tissue sample will be kept for the study (if possible).
• When a surgery for therapeutic purpose [eg. excision of one solitary brain metastasis, cementoplasty, bone fracture, decompression (intracranial hypertension, medullary compression…)] is performed, a tissue sample will be kept for the study (if possible).
• For patients with multiple metastatic sites, the most accessible and the less dangerous sites for the patient will be chosen for metastatic tumor sampling.

For bone metastasis: no tumor sampling allowed.
For brain metastasis, no tumor sampling allowed except surgical ones collected for therapeutic or diagnostic purposes.

If the patient takes an anticoagulant or an antiplatelet agent, the end of the study will be decided by patient's doctor or cardiologist.
Patients receiving anticoagulants or antiplatelet agents with anti-vitamin K are relayed by subcutaneous anticoagulants at effective dose. These should be stopped 24 hours before and retaken again 48 hours after the biopsy. If patients can't have this relay, biopsy won't be performed.

Patients receiving anticoagulants by subcutaneous injection should stop this treatment 24 hours before and retake it 48 hours after biopsy.
If patients can't have this relay, biopsy won't be performed.
If the patient takes an antiplatelet agent (aspirine > 160 mg/j and clopidogrel in particular) according to the indication should be stop this treatment 5 days before and retake 24 hours after biopsy, or be relayed by a subcutaneous anticoagulant treatment according to the same

terms as described above. If patient can't stop the antiplatelet agent treatment or have a relay, the biopsy won't be performed.

Stops and/or substitutions must be notified on patient's medical record very clearly.

NB : no peri-tumoral tissue and lymph nodes samples need to be collected for sarcoma cancer patients

4.1.2 Blood sampling

For this research study, all patients included will be asked to donate blood samples at different time points as indicated in sections 3.3.1 and 3.3.2
Specifically:

4.1.2.1 For newly diagnosed treatment-naïve ovarian cancer patients, triple negative breast cancer patients and Head and neck cancer patients upfront surgery

Blood samples must be collected at different times points:

1) at the time of primary surgery :

- 2 mL of blood collected for DNA extraction and sequencing analyses
- 5 ml of blood for serum
- 30 mL of blood for immune analyses and ctDNA

2) after surgery; 3) at disease recurrence before first cycle of chemotherapy or before radiotherapy; 4) before second cycle of chemotherapy or after radiotherapy and 5) at disease progression :

- 5 ml of blood for serum
- 30 mL of blood for immune analyses and ctDNA

In case of no recurrence, blood samples must be collected at six months after surgery (+/- 1 month) then at disease recurrence before first cycle of chemotherapy or before radiotherapy; before second cycle of chemotherapy or after radiotherapy and at a subsequent disease progression

- 5 ml of blood for serum
- 30 mL of blood for immune analyses and ctDNA

The risk of anemia is weak (around 1% of patients according to our previous experience). Nevertheless, hemoglobin levels, hematocrit and erythrocytes counts will be monitored at each blood withdrawal and in case of anemia (hemoglobin decreased by 15% or under 80 g/l) blood samples collection will be stopped and anemia will be monitored by standard care until normalization.

4.1.2.2 For newly diagnosed treatment-naïve cervical cancer patients

Blood samples must be collected at different times points:

1) at the time of primary surgery or before the beginning of chemoradiotherapy:

- 2 mL of blood collected for DNA extraction and sequencing analyses
- 20 mL of blood for immune analyses and ctDNA

2) after chemoradiotherapy (2 months after the end of treatment); 3) at disease recurrence before first cycle of chemotherapy; 4) before second cycle of chemotherapy and 5) at disease progression :

- 20 mL of blood for immune analyses and ctDNA

In case of no recurrence, blood samples must be collected at six months after the end of chemoradiotherapy (+/- 1 month) then at disease recurrence before first cycle of chemotherapy; before second cycle of chemotherapy and at a subsequent disease progression

- 20 mL of blood for immune analyses and ctDNA

The risk of anemia is weak (around 1% of patients according to our previous experience). Nevertheless, hemoglobin levels, hematocrit and erythrocytes counts will be monitored at each blood withdrawal and in case of anemia (hemoglobin decreased by 15% or under 80 g/l) blood samples collection will be stopped and anemia will be monitored by standard care until normalization.

4.1.2.3 For newly diagnosed treatment-naïve ovarian cancer patients, triple negative breast cancer patients before neoadjuvant chemotherapy

Blood samples must be collected at different times points:

1) before neadjuvant therapy :

- 2 mL of blood collected for DNA extraction and sequencing analyses
- 5 ml of blood for serum
- 30 mL of blood for immune analyses and ctDNA

2) during neadjuvant therapy (post cycle 1); 3) at the time of surgery; 4) at disease recurrence before first cycle of chemotherapy or before radiotherapy; 5) before second cycle of chemotherapy or after radiotherapy and 6) at a subsequent disease progression :

- 5 ml of blood for serum
- 30 mL of blood for immune analyses and ctDNA

In case of no recurrence, blood samples must be collected at six months after surgery (+/- 1 month) then at disease recurrence before first cycle of chemotherapy or before radiotherapy; before second cycle of chemotherapy or after radiotherapy and at a subsequent disease progression

- 5 ml of blood for serum
- 30 mL of blood for immune analyses and ctDNA

The risk of anemia is weak (around 1% of patients according to our previous experience). Nevertheless, hemoglobin levels, hematocrit and erythrocytes counts will be monitored at each blood withdrawal and in case of anemia (hemoglobin decreased by 15% or under 80 g/l) blood samples collection will be stopped and anemia will be monitored by standard care until normalization.

Note : the kind of tube is described in the "SCANDARE Study Procedures Manual".

4.1.2.4 For newly diagnosed treatment-naïve sarcoma cancer patients upfront surgery

Blood samples must be collected at different times points:

1) at the time of primary surgery:

- 22 mL of blood collected for DNA extraction, sequencing analyses, immune analyses, ctDNA

2) at disease recurrence before first cycle of systemic treatment ; 3) before third cycle of systemic treatment and 4) at disease progression :

- 20 mL of blood for immune analyses and ctDNA

In case of no recurrence, blood samples must be collected at six months after surgery (+/- 1 month) then at disease recurrence before first cycle of systemic treatment; before third cycle of chemotherapy and at a subsequent disease progression

- 20 mL of blood for immune analyses and ctDNA

The risk of anemia is weak (around 1% of patients according to our previous experience). Nevertheless, hemoglobin levels, hematocrit and erythrocytes counts will be monitored at each blood withdrawal and in case of anemia (hemoglobin decreased by 15% or under 80 g/l) blood samples collection will be stopped and anemia will be monitored by standard care until normalization.

4.1.2.5 For newly diagnosed treatment-naïve sarcoma cancer patients **before systemic treatment**

Blood samples must be collected at different times points:

1) before systemic treatment:

- 22 mL of blood collected for DNA extraction, sequencing analyses, immune analyses,ctDNA

Ensemble, prenons le cancer de vitesse.

2) during systemic treatment before cycle 3 (if no recurrence); 3) at disease progression :

- 20 mL of blood for immune analyses and ctDNA

The risk of anemia is weak (around 1% of patients according to our previous experience). Nevertheless, hemoglobin levels, hematocrit and erythrocytes counts will be monitored at each blood withdrawal and in case of anemia (hemoglobin decreased by 15% or under 80 g/l) blood samples collection will be stopped and anemia will be monitored by standard care until normalization.

Note : the kind of tube is described in the "SCANDARE Study Procedures Manual".

4.2 Tissue Sampling Management

Tumor, blood and lymph nodes samples labelling, storage and shipment will be described in the "SCANDARE Study Procedures Manual" provided by Institut Curie at the beginning of the Study.

5. MOLECULAR, TUMOR MICROENVIRONMENT AND IMMUNE ANALYSES

Please refer to table 2 in appendix 2

5.1 Molecular analysis on the tumor biopsies

- Retrospective exome, RNA and targeted sequencing analyses for will be performed on 400 patients.
- Biopathology approaches for the validation of molecular alterations detected by molecular analyses : exome, dedicated NGS will be done
- Retrospective ultra-deep sequencing to assess tumor heterogeneity will be performed on a small subset of patients for whom circulating tumor DNA analyses at different time point will show ne novo mutation.

5.2 Molecular analysis on circulating tumor DNA (ctDNA)

- Retrospective exome and targeted sequencing analyses for will be performed on 400 patients at different time point to assess temporal heterogeneity

5.3 Tumor MicroEnvironment (TME) analysis

- Detailed phenotypic analysis of the different components of the TME using various technologies will be used to evaluate tumor epithelial cells, endothelial cells, cancer associated fibroblast subsets and various immune cells types, namely, T, B and NK cells, macrophages, neutrophils and DC subsets.
- Systemic description of the soluble TME will be performed through the Luminex-based analysis of tumor-conditioned supernatants that reflect the composition of the soluble TME of human fresh primary tumors.

5.4 Immune analysis

- Whole blood phenotype will be performed on fresh blood samples to characterize the proportion of memory and naïve T cells, effector and memory subsets, innate like T cells, regulatory T cells, B cells, NK cell subsets, as well as different activation markers.
- Expression of PD-1 and other checkpoint molecules on the immune cells with a focus on the effector (CD127loCD25negCD27neg) CD4 T cells and CD27neg CD8 T cells that are increased in cancer patients will be done.

6. STUDY DESIGN

This research study involving cancer patients allows the collection of blood and tissue samples at different time points of treatment, as outlined in Section 3.3.1 and 3.3.2.

All visits and treatments are part of standard of care provided to patients.

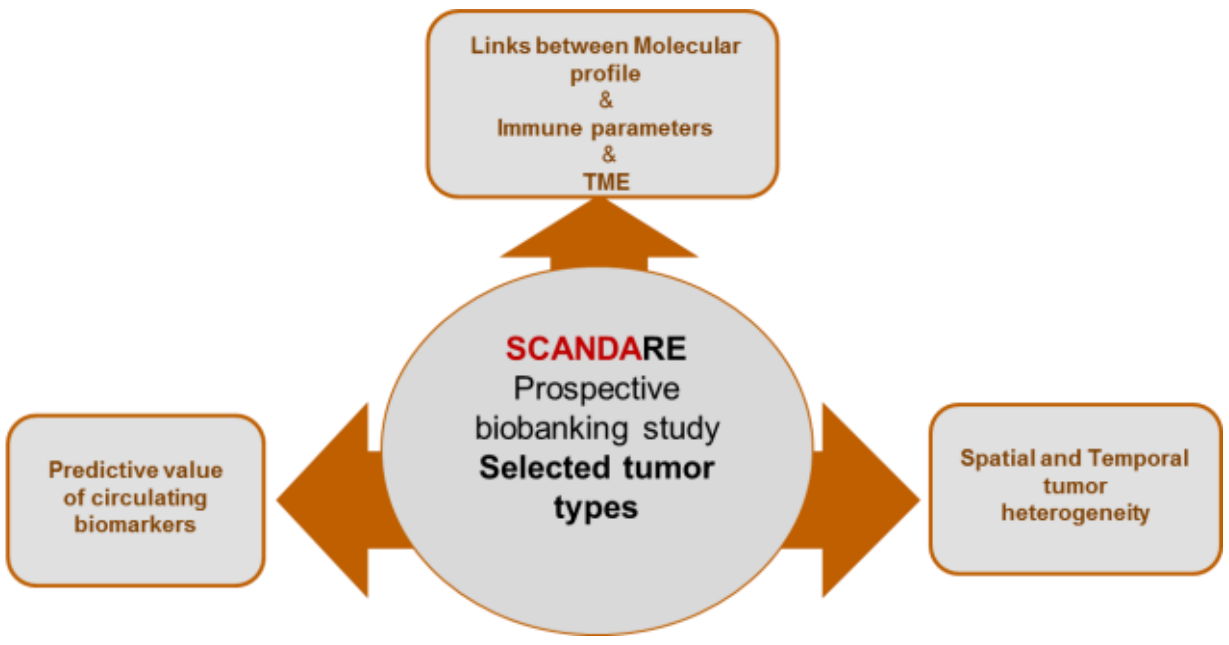

Consent form will have to be signed before any procedure.

For patients undergoing primary surgery, only additional blood samples will be taken from the patient at specific time points.

Patients undergoing neoadjuvant or exclusive chemo/radiotherapy will need to undergo a tumor biopsy in the frame of the study, along with blood samples.

7. RECOMMENDATIONS FOR INDIVIDUAL TREATMENT OPTIONS

Not applicable

8. SAFETY ASSESSEMENT

8.1 General Definition

A serious adverse event is any undesirable medical event that:

- results in death
- is life-threatening
- requires in-patient hospital stay or prolongation thereof
- results in persistent significant disability
- induces a congenital anomaly / birth defect or abortion
- is medically relevant

The terms disability and incapacity match with all physical/psychological temporary or permanent handicap, clinically significant with consequences on the physical activities and/or the quality of life of the patient. The definition of serious adverse event (experience) also includes important medical event. Medical and scientific judgment should be exercised in deciding whether expedited reporting is appropriate in other situations, such as important medical events (clinical or biological events) that may not be immediately life-threatening or result in death or hospitalization but may jeopardize the patient or may require intervention to prevent one of the other outcomes listed in the definition above. These should also usually be considered serious. Examples of such events are intensive treatment in an emergency room or at home for allergic bronchospasm; blood dyscrasias or convulsions that do not result in hospitalization; occurrence of a second cancer; pregnancy; or development of drug dependency or drug abuse.

Are not considered serious adverse events:

- a hospitalization < 24 hours,
- a hospitalization that was planned prior to the trial's beginning and/or scheduled by the protocol (biopsy, chemotherapy…).

Only serious adverse event related to tumor biopsies and blood samples will be reported.

8.2 Intensity Criteria

The intensity criteria must not be confused with the seriousness criterion that is utilized to define the obligations of declaration.
The events intensity is to be estimated according to the NCI CTC-AE version 4.03 classification. The intensity of the AE not listed in this classification will be evaluated according to the following scale:

Mild (grade 1): does not affect the usual daily activity of the patient
Moderate (grade 2): perturbs the usual daily activity of the patient
Severe (grade 3): hampers the usual daily activity of the patient
Life threatening or disabling (grade 4): requires intensive cares / is life-threatening
Lethal (grade 5): results in death

8.3 Reporting Serious Adverse Event

All Serious Adverse Events, **suspected to be related to the study specific** procedures to the informed consent form signature and within 30 days after the last study procedure, must be reported to the R&D UNICANCER Safety Office.

The SAE declaration must be faxed to the R&D UNICANCER Safety Office within 24 working hours of the initial observation of the event using a **SAE notification form**:

R&D UNICANCER – Pharmacovigilance

Phone: +33-1-44-23-04-16 – Fax: +33-1-44-23-55-70

Email: PV-RD@unicancer.fr

All provided additional documents must be made anonymous. More information might be requested by the R&D UNICANCER Safety Office.

8.4 Serious Adverse Event Follow-up

The investigator is responsible for the appropriate medical follow-up of the patients until the recovery, the stabilization or the death of the patient. **If required, follow-up might be maintained beyond the end of the protocol.**

9. STATISTICAL CONSIDERATIONS

9.1 Sample size and power considerations

It is an exploratory study without any hypothesis to test.
Patients will be enrolled in 5 different cohorts: one cohort for patients with triple negative breast cancer, one cohort for patients with ovarian cancer, one cohort for patients with head and neck cancer, one cohort for patients with cervical cancer and one cohort for patients with angiosarcoma or uterine sarcoma.

Based on the feasibility of the sampling, on financial and logistical constraints and on enrollment possibilities, a total of 500 patients with breast or ovarian cancers, 300 patients with head and neck cancer,200 patients with cervical cancer and 50 patients with angiosarcoma or uterine sarcoma are enrolled in order to establish the correlations between the different immune or molecular parameters and clinicobiological features.

The primary endpoint of the study is to evaluate association between new molecular and/or immunological biomarkers with clinical and biological features of the tumors.

Two-hundred patients will allow us to highlight the presence of a biomarker in the population with a precision level estimated in the table below:

% of patients with the biomarker	precision level	95% CI
5%	± 3.1%	[2.7 ; 9.0]
10%	± 4.2%	[6.6 ; 14.9]
15%	± 4.9%	[10.7 ; 20.6]
20%	± 5.5.%	[15.0 ; 26.1]
25%	± 6.0%	[19.5 ; 31.4]
30%	± 6.3%	[24.1 ; 36.7]
35%	± 6.6%	[28.7 ; 41.8]
40%	± 6.7%	[33.5 ; 46.9]
45%	± 6.8%	[38.3 ; 51.9]
50%	± 6.9%	[43.1 ; 56.9]

With 200 patients, the power of association test performed by Chi-square test will reach between 85% and 99% depending on the observed effect size; the power to observe a medium effect size will be 85 % while it will reach 99% to highlight a large effect size.

Angiosarcoma or uterine sarcoma are rare diseases. The number of patients enrolled is based on the limited number of cases diagnosed each year at the Institut Curie. The analyses will be purely exploratory analyses with no prespecified power.

9.2 Statistical Analysis

The baseline features of the patients will be reported. Continuous variables will be described as mean and standard deviations and compared with the use of the t test or the Mann-Whitney Test when appropriate. If necessary the continuous biomarkers values will be converted to log values.

Categorical variables will be presented as numbers and percentages and evaluated with the Chi-square test or Fisher's exact test.

Correlation between continuous biomarkers will be calculated using the Pearson product-moment correlation coefficient.

The recurrence free survival will be defined as the time from the date of inclusion to the first documentation of local, regional or distant disease or death, whichever occurs first. The patients who will not experiment any event will be censored at their date of last follow-up.The recurrence free survival will be estimated using the Kaplan-Meier method and survival curves will be compared using a log-rank test.

If appropriate, biomarkers and/or clinical features will be included in a multivariate stepwise procedure using the Cox model.

A pvalue less than 0.05 will be considered to be statistically significant

10. DATA PROTECTION AND CONFIDENTIALITY MANAGEMENT

Study data are processed in accordance with the methodology of reference 001 published by the French CNIL. Institut Curie has ensured the compliance to this methodology before submitting the study to the CPP and information to ANSM.

Data analysis will be performed by the biostatistics service of Institut Curie under the supervision of Alexia Savignoni (statistician) and the principal coordinators of the project.

Until the trial results are published, the investigator is responsible for insuring the confidentiality of the totality of the information, handled by him and all other individuals involved in the course of the trial. This obligation holds neither for the information that the investigator may communicate to the patients within the context of the trial nor for the already published information.

Nevertheless, in conformity with the article R 5121-13 of the Public Health Code, both the center and the investigator may communicate information relative to the trial:

- to the Health Minister,
- to the public health inspectors,
- to the ANSM General Director and inspectors.

11. QUALITY ASSURANCE AND QUALITY CONTROL

11.1 Data Safety Monitoring Board (DSMB)

As this study do not interferes with current standard of care, data safety monitoring board is not planned.

11.2 Quality control

In order to guaranty the authenticity and credibility of the data in accordance with the Good Clinical Practices, the sponsor will set up an assurance quality program that includes:

- management of the trial according to the Institut Curie procedures,
- quality control of the data provided by the investigation site is performed by the study monitor whose role is to match and check the consistency of the data reported in the Case Report Form with respect to the source-documents, possible audit and inspection of investigational sites, centralized review of selected criteria of the protocol.

12. ETHICAL AND REGULATORY ISSUES

The clinical trial must be conducted in accordance with:

- the principles of ethics as stated in the last version in use of the Declaration of Helsinki,
- the Good Clinical Practices defined by the International Conference on Harmonization (ICH–E6, 17/07/96),
- the European directive 2001/20/CE on the conduct of clinical trials,
- the law on ‘informatics and freedom’ (Informatique et Libertés n° 78-17) of January 6th, 1978 modified notably by the law n° 2016-41 of January 26th, 2016 of modernization of our Health System

- bioethic law n° 2004-800 of August 6, 2004.

- the law n° 2012-300 of March 5th 2012 relating to the researches involving the human person

- regulation of the European Parliament and of the Council of 27 April 2016 on the protection of natural persons with regard to the processing of personal data and on the free movement of such data

12.1 Ethics Committee

Before starting a biomedical research on human patients, the sponsor must submit its project to the opinion of one of the competent Ethics Committee in the region where the principal investigator is practicing.

A request for opinion on the biomedical research project is addressed to the CPP by the sponsor.

Request of substantial modifications to initial projects are submitted for the CPP's opinion by the sponsor as well.

12.2 Competent authority

Before carrying out or letting a study to be carried out on its behalf, the sponsor must file a request with the competent authority.

Request of substantial modifications to initial projects are submitted for the competent authority's opinion by the sponsor as well.

12.3 Information and consent of the participants

Prior to carrying out biomedical research on human patients, a free and written informed consent form must be signed by each individual participating in the trial after she has been informed by the investigator during a physician-patient consult and after sufficient time for reflection has been allowed.

Information given to the trial participants must cover all of the elements defined by the public health law of August 9th, 2004 and must be written in a simple and comprehensible patient-appropriate manner. Once the participant is acquainted with the information, she/he must sign all the pages of the information sheet. The original sheet will be kept in the investigator's folder and the duplicate copy will be returned to the participant.

The consent form must be dated and signed by both the participant in research and the investigator. The original document is archived by the investigator; a copy is given to the research participant.

The information sheet and informed consent form must be associated within the same document to insure that the whole information is given to the participant.

12.4 Responsibilities of the sponsor

The sponsor (Institut Curie) of the trial is the natural or moral person that takes the initiative of conducting study on human patients, and is therefore accountable for the research management and for verifying the financing schedule.

The main sponsor responsibilities are:

- to subscribe a civil-responsibility insurance,
- to register the trial in the ANSM data base,
- to request the opinion of the Committee for the Protection of Persons (CPP) on the initial project and the substantial amendments,
- to file the demand of authorization for the initial project and all substantial amendments with the competent authorities,
- to provide information on the trial to the heads of the health care centers, the appropriate investigators and the pharmacists (if applicable),
- to declare to the competent authorities and other regulatory agencies, any suspicion of unexpected serious adverse events and communicate the information to the CPP and the investigators of the trial,
- the annual declaration of the security report to the competent authorities and the CPP,
- the declaration of the beginning and the end of the trial to the competent authority,
- editing the final report on the trial,
- communicating the information on the trial's results to the competent authority, the CPP and the research participants,
- archiving the trial's essential documents in the sponsor folder for a minimal duration of 15 years after the research is ended.

12.5 Responsibilities of the investigators

Investigator responsibilities are set out in the ICH Guideline for GCP (good clinical practice) and the applicable regulatory requirements.

The main investigator of each concerned healthcare center commits to conducting the clinical trial in compliance with the protocol that has been approved by the CPP and the competent authority.

The investigator must not bring any modification to the protocol without having obtained written authorization of the sponsor and the proposed modifications have been authorized by the ethics committee and the competent authority.

It is the responsibility of the principal investigator:

- to provide the sponsor with its own curriculum vitae and co-investigators' curriculum vitas,
- to adhere to the document/records retention procedures by signing the protocol,

- to identify the members of its team that participate in the trial and to define their responsibilities,
- to insure patient recruitment after the sponsor has issued its authorization.

It is the responsibility of each investigator:

- to collect the informed consent form, dated and signed personally by each individual research participant before any selection procedure specific to the trial may start,
- to regularly fill in the case report form (CRF) for each patient included in the trial and to allow the CRA (Clinical Research Associate) mandated by the sponsor to have direct access to the source-documents in order to validate the data collected and resolved any queries
- to date, correct and sign the corrections brought to the Case Report Form for each patient included in the trial,
- to accept regular visits of the study monitor and possibly the auditors mandated by the sponsor or the inspectors of the legal competent authorities,
- archiving the trial's essential documents for a minimal duration of 15 years after the research is ended.

Until the trial results are published, the investigator is responsible for insuring the confidentiality of the totality of the information, handled by him and all other individuals involved in the course of the trial. This obligation holds neither for the information that the investigator may communicate to the patients within the context of the trial nor for the already published information.

The investigator(s) must ensure that the records and documents pertaining to the conduct of the study and the distribution of the study treatment are complete, accurate, filed and retained.

12.6 Federation of the Patient Committees for Clinical Research in Cancerology

The creation of the Federation of the Patient Committees for Clinical Research in Cancerology (FCPRCC) is the initiative of the UNICANCER and the LNCC (National League for Treating Cancer). Its dedicated task is to make a second read of the clinical trial protocols in cancerology. The patient committees' federation is coordinated by the BECT (Clinical and Therapeutic Trials Offices), a department of the UNICANCER. It gathers both the patient committees of the LNCC and other health care centers. It commits to rereading the protocol and proposing improvements dealing principally with the quality of the letter of information to the patients, the setting up of a treatment and monitoring plan, suggesting measures aimed at ameliorating the comfort of the patients.

13. PUBLICATION OF RESULTS

All information resulting from this trial is considered to be confidential, at least until first communication and after appropriate analysis and checking has been completed by the sponsor, the principal investigator and the statistician of the trial.

SCANDARE is a biobanking study; the material collected during this study will be used for molecular and immune analyses on the tumor and its microenvironment. The work performed will be in collaboration between different translational and clinical teams at Institut Curie.

Consequently, the first author and writer of the publication will be the principal investigator or another collaborator, according to the number of recruited patients and the scientific work performed regardless of the importance of the cooperating group they belong to. The statistician and a sponsor representative will be cited as well as all the individuals who have taken part in carrying out these sub-studies and a sponsor representative.

14. FUNDING AND INSURANCE

14.1 Funding

This work is supported by the grant ANR-10-EQPX-03 and ANR10-INBS-09-08 from the Agence Nationale de la Recherche and by the Canceropôle Ile-de-France.

14.2 Insurance

Sponsor will provide insurance for the clinical trial.

15. REFERENCES

1. Shekhar, M. P., Santner, S., Carolin, K. A. & Tait, L. Direct involvement of breast tumor fibroblasts in the modulation of tamoxifen sensitivity Am J Pathol 170, 1546-1560 (2007).
2. Wang W, Li Q, Yamada T, Matsumoto K, Matsumoto I, Oda M, Watanabe G, Kayano Y, Nishioka Y, Sone S, Yano S. Crosstalk to stromal fibroblasts induces resistance of lung cancer to epidermal growth factor receptor tyrosine kinase inhibitors. Clin Cancer Res. 2009 Nov 1;15(21):6630-8
3. Johansson AC, Ansell A, Jerhammar F, Lindh MB, Grénman R, Munck-Wikland E, Östman A, Roberg K. Cancer-associated fibroblasts induce matrix metalloproteinase-mediated cetuximab resistance in head and neck squamous cell carcinoma cells. Mol Cancer Res. 2012 Sep;10(9):1158-68.
4. Straussman R, Morikawa T, Shee K, Barzily-Rokni M, Qian ZR, Du J, Davis A, Mongare MM, Gould J, Frederick DT, Cooper ZA, Chapman PB, Solit DB, Ribas A, Lo RS, Flaherty KT, Ogino S, Wargo JA, Golub TR. Tumour micro-environment elicits innate resistance to RAF inhibitors through HGF secretion. Nature. 2012 Jul 26;487(7408):500-4.
5. Hodi FS, O'Day SJ, McDermott DF, Weber RW, Sosman JA, Haanen JB, Gonzalez R, Robert C, Schadendorf D, Hassel JC, Akerley W, van den Eertwegh AJ, Lutzky J, Lorigan P, Vaubel JM, Linette GP, Hogg D, Ottensmeier CH, Lebbé C, Peschel C, Quirt I, Clark JI, Wolchok JD, Weber JS, Tian J, Yellin MJ, Nichol GM, Hoos A, Urba WJ. Improved survival with ipilimumab in patients with metastatic melanoma. N Engl J Med. 2010 Aug 19;363(8):711-23.
6. Topalian SL, Hodi FS, Brahmer JR, Gettinger SN, Smith DC, McDermott DF, Powderly JD, Carvajal RD, Sosman JA, Atkins MB, Leming PD, Spigel DR, Antonia SJ, Horn L, Drake CG, Pardoll DM, Chen L, Sharfman WH, Anders RA, Taube JM, McMiller TL, Xu H, Korman AJ, Jure-Kunkel M, Agrawal S, McDonald D, Kollia GD, Gupta A, Wigginton JM, Sznol M.Safety, activity, and immune correlates of anti-PD-1 antibody in cancer. N Engl J Med. 2012 Jun 28;366(26):2443-54.
7. Topalian SL, Sznol M, McDermott DF, Kluger HM, Carvajal RD, Sharfman WH, Brahmer JR, Lawrence DP, Atkins MB, Powderly JD, Leming PD, Lipson EJ,

Puzanov I, Smith DC, Taube JM, Wigginton JM, Kollia GD, Gupta A, Pardoll DM, Sosman JA, Hodi FS. Survival, durable tumor remission, and long-term safety in patients with advanced melanoma receiving nivolumab. J Clin Oncol. 2014 Apr 1;32(10):1020-30.

16. APPENDIX

APPENDIX 1 : Patients samples

Patients population	Description	Fresh Tumor samples + normal surrounding tissue[2] *(if possible)* + tumor scar tissue[1] *(if possible)*	Tumor-draining Lymph nodes	Fixed Tumor samples + normal surrounding tissue[2] *(if possible)* + tumor scar tissue[1] *(if possible)*	Frozen Tumor samples + normal surrounding tissue[2] *(if possible)* + tumor scar tissue[1] *(if possible)*	Blood	
						EDTA Tubes	Dry Tubes
**Ovarian cancer patients treated with up-front surgery**	Surgical specimen (Celioscopy or Laparotomy)	>2cm*	Whenever possible	2 Blocs	2 frozen tumors	****2 mL**→ DNA Extraction and sequencing analyses **30 mL**→ immune analyses and ctDNA	**5 mL**→ serum
**Ovarian cancer patients treated with neoadjuvant therapy** (minimum 50)	Pretreatment biopsy	Fresh tumor required	Whenever possible	2 Blocs	2 frozen tumors	****2 mL**→ DNA Extraction and sequencing analyses **30 mL**→ immune analyses and ctDNA	**5 mL**→ serum
	Surgical specimen post- NAC	>2cm*	Baseline if possible	2 Blocs	2 frozen tumors	****2 mL**→ DNA Extraction and sequencing analyses **30 mL**→ immune analyses and ctDNA	**5 mL**→ serum
**TN breast cancer patients treated with up-front surgery** (minimum 50)	Surgical specimen	>2cm*	Whenever possible	2 Blocs	2 frozen tumors	****2 mL**→ DNA Extraction and sequencing analyses **30 mL**→ immune analyses and ctDNA	**5 mL**→ serum
**TN breast cancer patients treated with neoadjuvant therapy** (minimum 50)	Pretreatment biopsy	Fresh tumor required	Whenever possible	2 Blocs	2frozen tumors	****2 mL**→ DNA Extraction and sequencing analyses **30 mL**→ immune analyses and ctDNA	**5 mL**→ serum
	Surgical specimen post- NAC	>2cm*	Whenever possible	2 Blocs	2frozen tumors	****2 mL**→ DNA Extraction and sequencing analyses **30 mL**→ immune analyses and ctDNA	**5 mL**→ serum

**Head and Neck cancer patients treated with up-front surgery** (minimum 50)	Surgical specimen	>2cm*	Whenever possible	2 Blocs	2 frozen tumors	****2 mL**→ DNA Extraction and sequencing analyses **30 mL**→ immune analyses and ctDNA	**5 mL**→ serum
Cervical cancer patients with upfront surgery (stage Ia-IIa1)	Surgical specimen	NA	Whenever possible	2 Blocs	2 frozen tumors	****2 mL**→ DNA Extraction and sequencing analyses **20 mL**→ ctDNA	**NA**
Cervical cancer patients with upfront surgery (stage≥ IIa2)	Biopsy	NA	Whenever possible	2 Blocs	2 frozen tumors	****2 mL**→ DNA Extraction and sequencing analyses **20 mL**→ ctDNA	**NA**
Sarcoma cancer patients treated with up-front surgery	Surgical specimen	>2cm*	NA	2 Blocs	1 frozen tumor	**20-22 mL**→ ctDNA, DNA Extraction and sequencing analyses	**NA**
Sarcoma cancer patients treated with systemic treatment	Pretreatment biopsy	Fresh tumor required	NA	2 Blocs	1 frozen tumor	**20-22 mL**→ ctDNA, DNA Extraction and sequencing analyses	**NA**

** just before surgery or Neoadjuvant Therapy

* possibility to use multiple fragments

[1] for Ovarian and Breast cancer patient before neoadjuvant chemotherapy only

[2] not requested for Sarcoma cancer patient

APPENDIX 2 : Techniques used per sample

Type of sample	NGS (WES/RNAseq or targeted NGS) or single cell sequencing	Ultra-deep seq	RNA-Seq	IHC	Luminex	Flow Cytometry	Other techniques
**Fresh Tumor samples + Normal surrounding tissue (*if possible*[2]) + tumor scar tissue *(if possible)* [1]**	**X**				**X**	**X**	**If possible and frozen fragment remaining→ Cell lines establishment**
**Tumor- draining Lymph nodes**	**X**			**X**		**X**	**Cell lines establishment**
**Fixed Tumor samples + Normal surrounding tissue *(if possible*[2]*)* + tumor scar tissue *(if possible)* [1]**		**X**		**X**			
**Frozen Tumor samples + Normal surrounding tissue *(if possible*[2]*)* + tumor scar tissue *(if possible)* [1]**	**X**		**X**	**X**			
**Plasma ctDNA**	**X**						X
**Blood**	**X**				**X**	**X**	

[1] for Ovarian and Breast cancer patient before neoadjuvant chemotherapy only

[2] not requested for Sarcoma cancer patient

APPENDIX 3 - Toxicity criteria (CTCAE)

http://ctep.cancer.gov/

Common Terminology Criteria for Adverse Events v4.03 (CTCAE)
