# Supplementary material for: Multiregion profiling of genomic and transcriptional heterogeneity in head and neck squamous-cell carcinoma
Source: ESMO Open. 2026 Sep 11;11(10):108367. doi: 10.1016/j.esmoop.2026.108367 (PMC13583575; doi:10.1016/j.esmoop.2026.108367)

Supplementary Fig. 1:

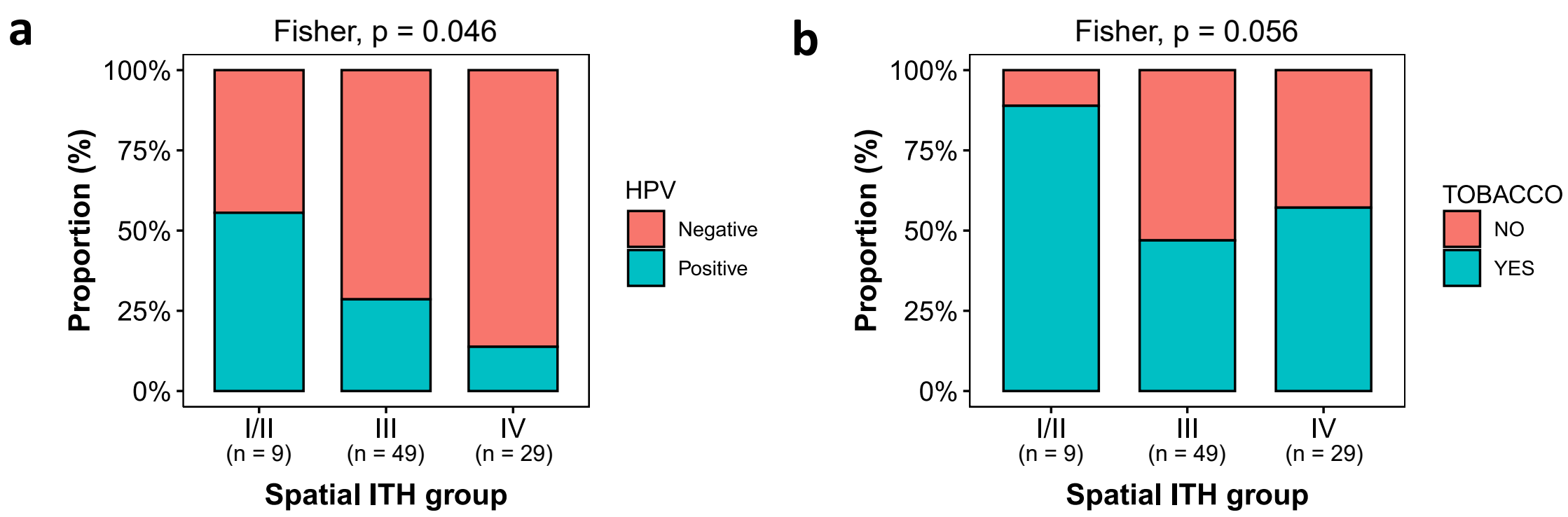

Supplementary Fig. 2:

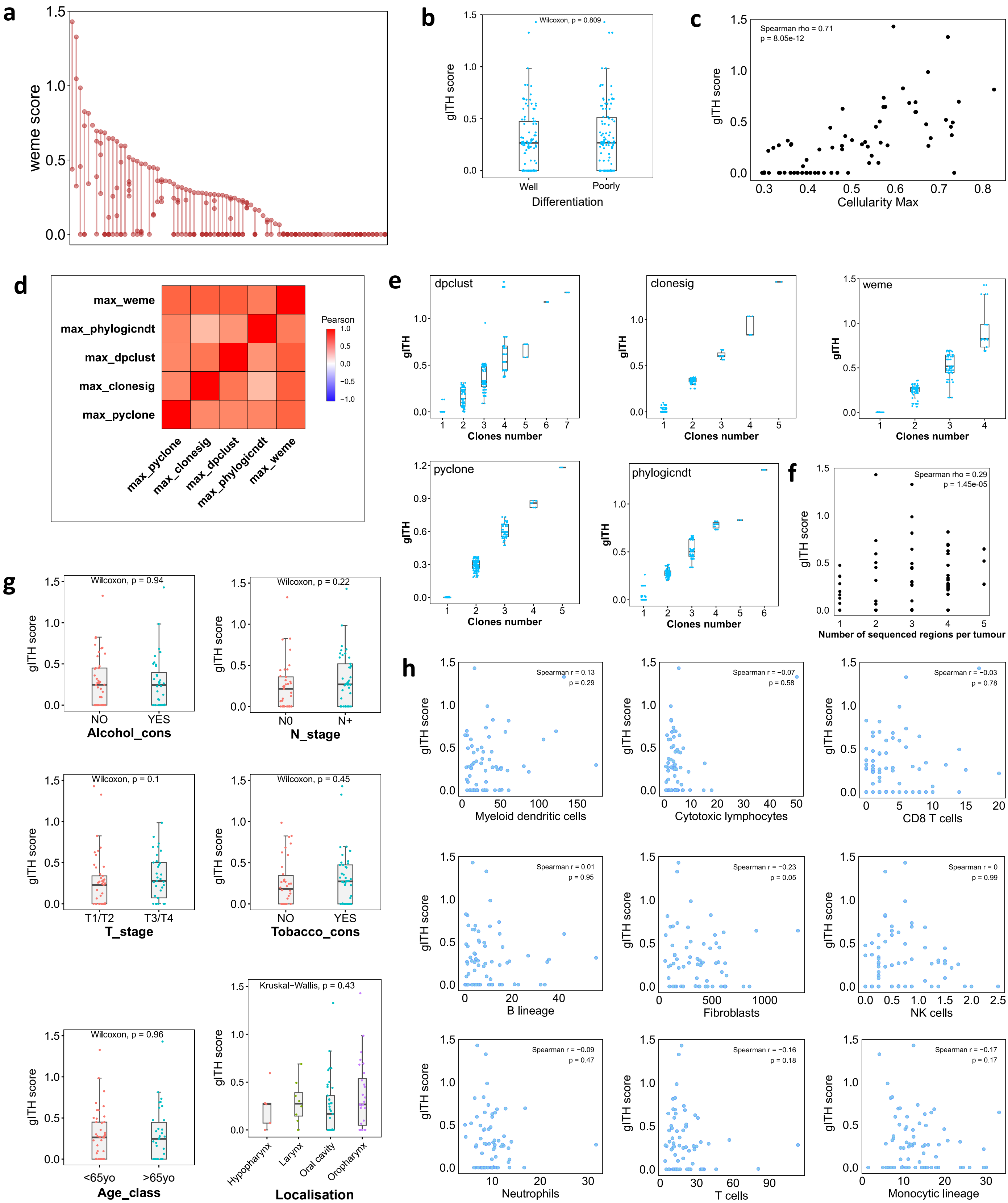

Supplementary Fig. 3:

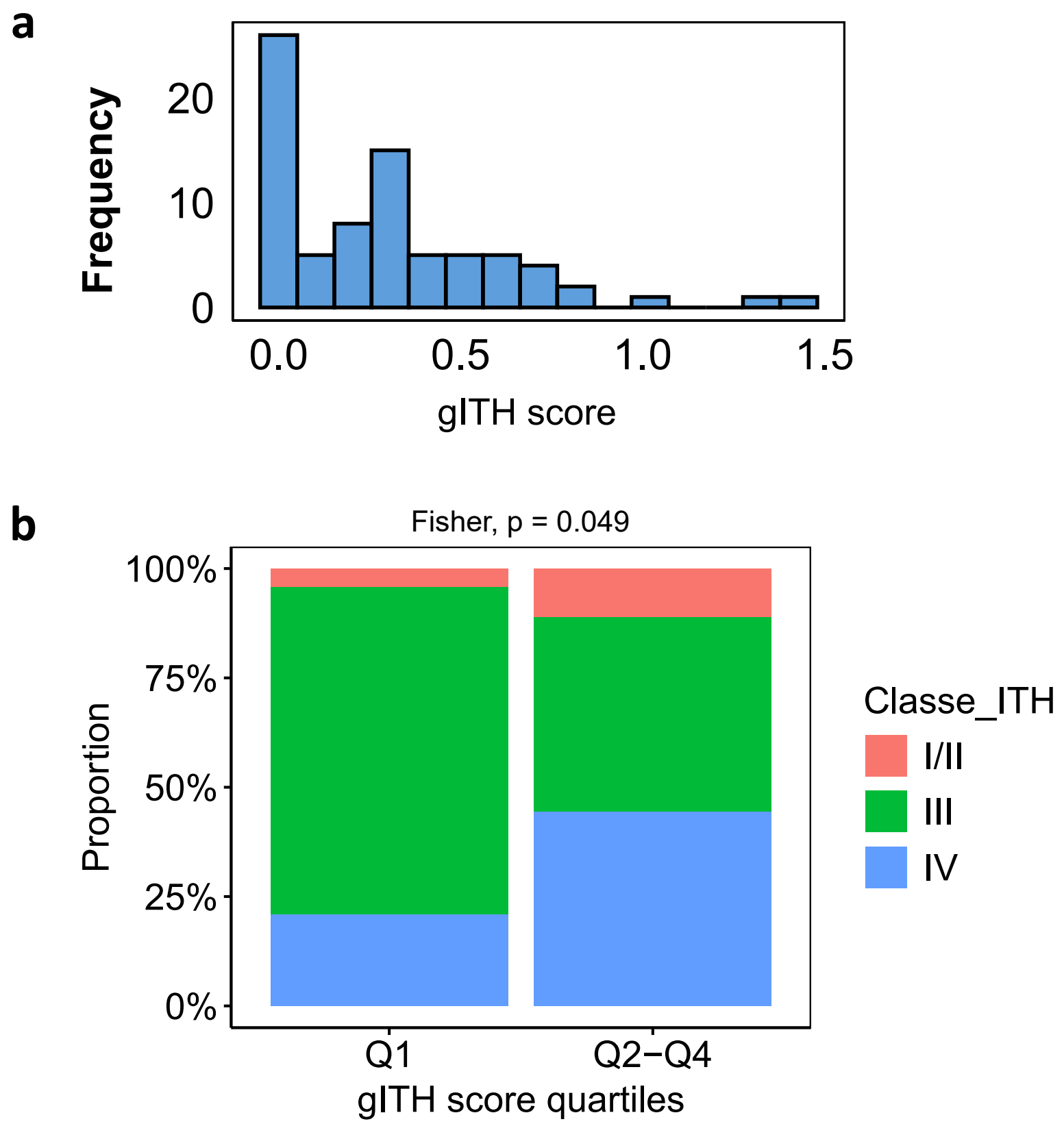

Supplementary Fig. 4:

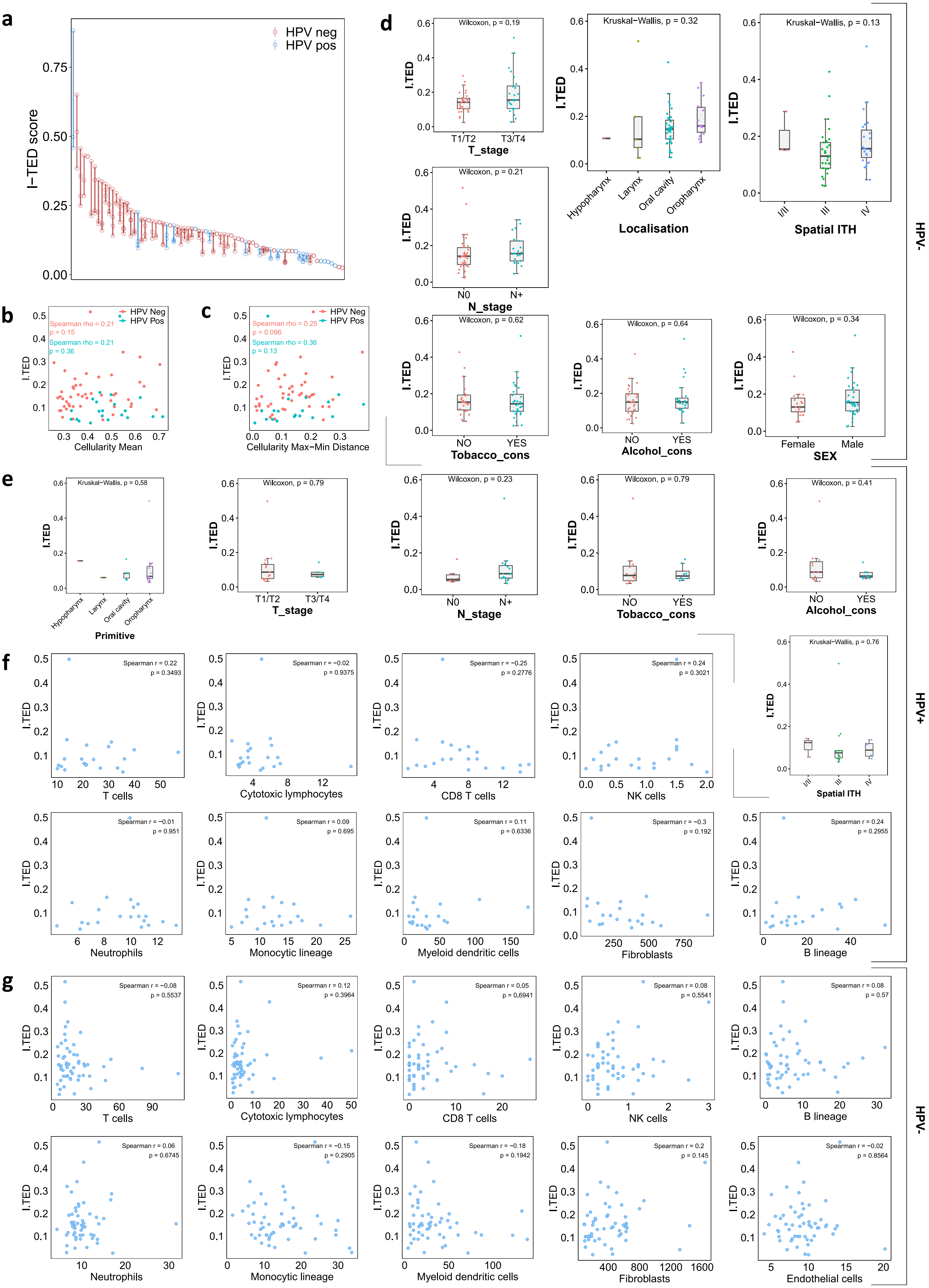

Supplementary Fig. 5:

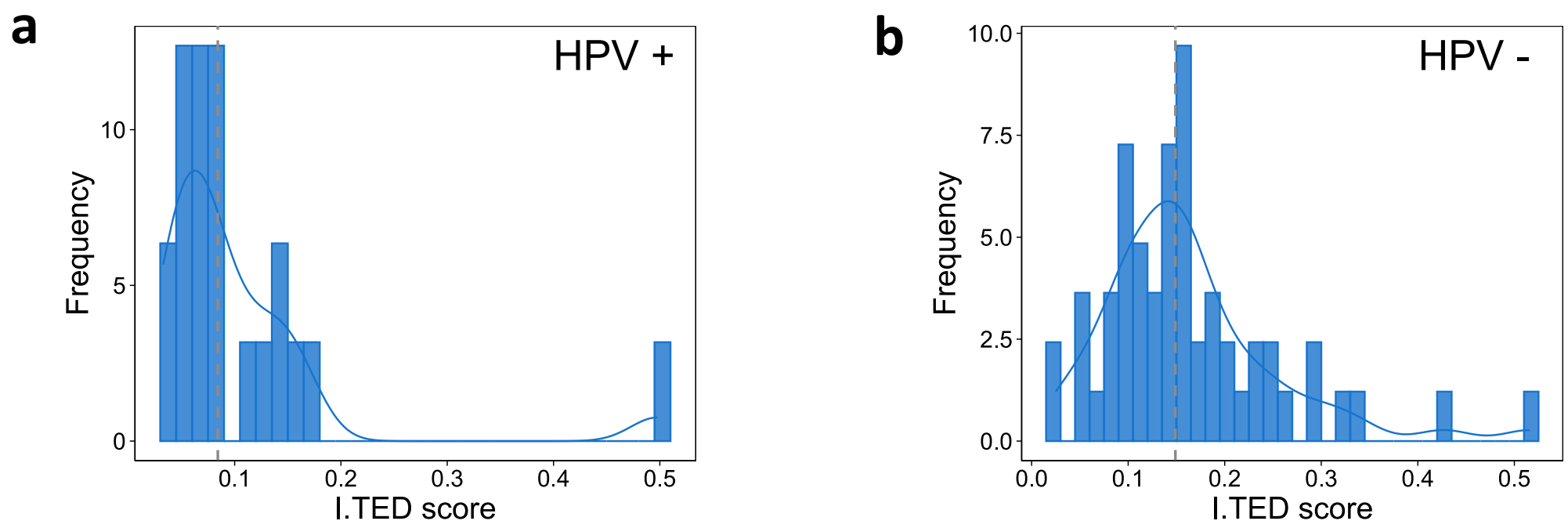

Supplement: Supplementary file 2 [file mmc4.pdf]
