# Supplementary material for: Multiregion profiling of genomic and transcriptional heterogeneity in head and neck squamous-cell carcinoma
Source: ESMO Open. 2026 Sep 11;11(10):108367. doi: 10.1016/j.esmoop.2026.108367 (PMC13583575; doi:10.1016/j.esmoop.2026.108367)
Supplement: Supplementary file 3 [file mmc5.docx]

**Patients and Methods**

**Patients and sample collection**

The SCANDARE biobanking clinical study (NCT03017573) is a prospective observational cohort designed, among other objectives, to investigate the relationship between molecular alterations in solid tumors and patients’ response to treatment or clinical outcomes. The present manuscript focuses on a subset of 90 patients enrolled in SCANDARE with early-stage head and neck squamous cell carcinoma (HNSCC), all treated with upfront curative-intent surgery at Institut Curie (Paris, France) between January 5, 2017, and July 4, 2019. All biological samples were collected in accordance with ethical standards, including the principles of Good Clinical Practice, the Declaration of Helsinki, and after obtaining written informed consent from all participants. The SCANDARE study was approved by a national ethics committee (CPP Île-de-France 3, approval number: 3440) and by the French National Agency for the Safety of Medicines and Health Products (ANSM, approval number: ID-RCB 2016-A01095-46). Formalin-fixed paraffin-embedded (FFPE) blocks from the primary resected tumour were obtained from all patients. For each tumour, one to five spatially distinct regions were delineated by a pathologist and subsequently analyzed using multiregional targeted DNA sequencing and bulk RNA sequencing. All patients received standard-of-care adjuvant therapy (e.g., radiotherapy with or without chemotherapy), with clinical follow-up performed according to disease stage and institutional guidelines. A three-year follow-up period was completed as part of routine clinical management.

**Pathological examination**

To assess intratumoral heterogeneity, a senior pathologist reviewed all available hematoxylin and eosin (H&E) stained slides for each primary tumour. Based on morphological criteria, the pathologist identified between one and five spatially distinct regions per tumour displaying phenotypically divergent differentiation status (well-differentiated vs poorly differentiated areas). Spatially distinct tumor regions were selected from the surgical specimen according to tumor cell enrichment, morphological features and tissue quality, in order to obtain representative sampling of the overall tumor while excluding poorly preserved or extensively necrotic areas. Each selected region was macroscopically delineated, and the following assessments and procedures were performed: measurement of tumour surface area, evaluation of tumour purity and stromal composition, HPV genotyping, and extraction of genomic DNA and total RNA. For each tumour, the mean proportions of well- and poorly differentiated regions were calculated. Based on this evaluation, we predefined four tumour classes: homogeneous tumors composed of more than 80% well-differentiated cells (class I) or more than 80% poorly differentiated cells (class II); and heterogeneous tumors, further subdivided according to the predominance of either well-differentiated (class III) or poorly differentiated (class IV) regions. These classes were used for subsequent correlation analyses with clinical features and outcomes, as detailed in the Results section.

**Nucleic acids extraction**

For each tumour sample, tissue sections of 6 μm thickness were obtained from a single FFPE tissue block. Tumour-rich areas were identified on one hematoxylin-eosin-stained slide and microdissected using a single-use blade on the remaining six slides for extraction. Samples underwent proteinase K digestion at 56°C for 3 days, followed by DNA extraction using the NucleoSpin kit (Macherey-Nagel, Düren, Germany) according to the supplier's protocols. DNA was quantified using the Qubit dsDNA HS assay kit (ThermoScientific, Wilmington, DE). RNA was extracted from the same tissue block using the High Pure FFPET RNA Isolation Kit (Roche Life Science, USA), in accordance with the manufacturer’s instructions. RNA concentration and purity were assessed via NanoDrop spectrophotometry (ThermoScientific, Wilmington, DE, USA).

**DNA sequencing and bioinformatic analysis**

Genomic DNA profiling of tumour samples was conducted on the DRAGON Dx (Detection of Relevant Alterations in Genes involved in Oncogenetics) platform, a pan-cancer diagnostic next-generation sequencing (NGS) panel comprising 571 genes (Supplementary table 2). The assay allows the detection of single nucleotide variants (SNV), small (<50 bp) insertions and deletions (indels), copy number variants (CNV), and microsatellites instability. Library preparation, sequencing and bioinformatic analysis were performed as described previously^1^. Quality-control criteria for sequencing data were: at least 1 million reads mapped, and at least 5% of targeted regions at a sequencing depth of 1000X or more. Sample purity (i.e., fraction of cancer cell) and copy-number alterations, including sub-clonal copy-number alterations, were determined using the FACETS tool^2^ (v0.6.2).

Only variants with a depth of coverage of at least 100X and allelic frequency of at least 10% were considered for downstream analysis. Strand bias was quantified by the Phred-scaled P value of the Fisher's exact test on the contingency table of the strand-specific distribution of reads supporting either the reference or alternative allele. SNV and indels with a score higher than 50 and 200, respectively, were filtered out. Variants with reported frequencies higher than 0.5% in any subpopulation in either gnomAD 26 (version 2.1.1) or Exome Sequencing Project (version ESP6500) databases were excluded from the analysis.

Genes were classified as tumour suppressors, oncogenes according to the literature and authoritative databases (cBioPortal^3^, OncoKB^4^, The Cancer Genome Atlas^5^, COSMIC^6^); genes with equivocal evidence for their specific role in carcinogenesis were classified assigned an “unknown” role. Variant pathogenicity was assessed using automated analysis using the OncoKB-annotator^7^ tool v3.3.2 and manual curation. Were classified as pathogenic: variants labelled as oncogenic, likely oncogenic, or predicted oncogenic by the OncoKB-annotator tool; truncating variants in tumour suppressor genes; variants with sufficient evidence for their oncogenic effect in the literature and databases in oncogenes and tumour suppressor genes (e.g., hotspot missense variants). Actionability of variant in HNSCC according to the ESMO Scale for Clinical Actionability of Molecular Targets (ESCAT) was determined as described previously^8^.

We systematically annotated pathogenic variants using the OncoKB and ESCAT frameworks to assess intratumoral heterogeneity in actionable biomarkers. Based on OncoKB, variants were classified as theranostic (*PIK3CA*, *EGFR*, and *TSC2*; Level I) or putative (*ARID1A*, *PTEN*, and *CDKN2A/B*; Level IV). Using ESCAT, variants were categorized as investigational (*EGFR* and *CDKN2A/B*; Tier IIA), hypothetical (*PIK3CA* and *PTEN*; Tier IIIA), or under development (*TP53*; Tier V). Variants without annotation in either OncoKB or ESCAT, but considered potentially actionable (*NOTCH1*, *POLE*, and *KMT2C*), were also reported.

**HPV typing**

HPV detection and genotyping were performed by real-time PCR using SYBR Green (Roche Diagnostics, Mannheim, Germany) and type-specific primers for HPV16, HPV18, HPV33, as well as primers targeting the human GAPDH gene. Amplification was carried out on a 7900HT Fast Real-Time PCR System (Applied Biosystems). For samples negative for HPV16, 18, and 33, HPV L1 amplicons were sequenced using the Sanger method with the GP5+ primer. HPV types were identified by aligning the resulting sequences with reference HPV genomes using NCBI’s BLAST tool.

**Genomic intra-tumour heterogeneity**

Single-sample subclonal deconvolution was performed using four primary algorithms: PyClone-Vi^9^ (v.0.1.1), DPClust^10^ (v2.2.8), PhylogicNDT^11^ (v1.0) and CloneSig^12^ (v1.0.1). In this context, clones were defined as distinct populations of cancer cells sharing a specific set of somatic genomic variants. These methods leverage nucleotide variant allele frequency and local copy-number state to infer the clonal architecture of each tumour sample, i.e. enumerate cancer cell clones and their cancer cell fraction (CCF), the fraction of the total cancer cell population they represent. Inputs for all algorithms were limited to high-confidence variants, as defined by the filtering criteria described above, and excluded variants located in regions affected by subclonal copy number alterations. Due to algorithmic limitations, CloneSig was applied only to single-nucleotide variants, excluding indels. Outputs of the four primary algorithms were passed to WeMe^13^, a meta-deconvolution algorithm that aggregates outputs from multiple tools to generate a robust description of clonal architecture for each sample. A single-sample numerical estimations of genomic intra-tumour heterogeneity (gITH) was derived using the Shannon index, an ecological diversity metric, using the following formula (for a sample with j clones) :

$$gITH= -\sum_{i=1}^{j} {CCF}_{i}\ln({CCF}_{i})$$

**RNA sequencing and bioinformatic analysis**

RNA library preparation was performed using the QuantSeq 3’ mRNA-Seq Library Prep Kit REV (Lexogen, Vienna, Austria) following the manufacturer’s instructions, and sequencing was carried out on a NovaSeq 6000 (Illumina Inc., San Diego, CA, USA). Raw sequencing data were processed using the Institut Curie RNA-seq pipeline v4.1 (DOI: 10.5281/zenodo.13744441), which leverages STAR^14^ for read alignment and quantification of gene expression levels. All differential gene expression and subsequent gene set enrichment analyses (GSEA) were performed using the following approach. Differential expression analysis was performed using the DESeq2^15^ pipeline (v.1.44.0) (design = ~ gITH or ~ tITH taken as a continuous variable) on raw counts, after filtering lowly expressed genes with filterByExpr (edgeR)^16^. Log2 fold changes were shrank with the apeglm method, and genes with adjusted p-value (FDR) < 0.05 were considered significantly differentially expressed. Hallmark gene sets were retrieved from MSigDB^17^ (msigdbr). Genes were ranked using the product of log2 fold change and –log10(p-value), and enrichment analyses were conducted using GSEA (for Hallmark sets) and gseGO (for Gene Ontology Biological Process terms), with a p-value cutoff of 0.05.

**I.TED score calculation**

I.TED calculation was done according to Carlos Martínez-Ruiz et al.^18^ across HPV positive or negative tumors samples separately on the R2 cohort. Quiclky, raw read counts were normalized using the median of ratios method applied in DESeq2 (v.1.44.0). Genes with more than 5 read counts in at least 20% of the samples (a total of 18,301 genes) were kept after filtering. Variant stabilizing transformation (VST) was performed on the normalized reads. For each region of a tumour, 1 minus the correlation distance between VST gene expression to all other regions in the same tumour was calculated for the top 500 most variable genes. The correlation distance was calculated using the function dcor() in the R package energy (v.1.7-12). Tumour level I.TED was calculated as the median I.TED of all regions.

**RNA-seq tumour microenvironment deconvolution**

Gene expression profiles (TPM) were aggregated at the patient level by averaging all the sequenced regions of the tumour. Immune cell populations were quantified using MCPcounter^19^ (v1.2.0) with ENSEMBL gene identifiers. Pearson correlation coefficients were then computed between immune scores and intra-tumour heterogeneity metrics, separately in HPV-positive and HPV-negative cohorts for transcriptional heterogeneity.

**Survival analysis**

Disease-free survival (DFS) (defined as the time from diagnosis to the first documented relapse - local, regional, or distant - or death from any cause, with patients without events censored at the last follow-up) and overall survival (OS) were analysed using Kaplan–Meier estimates and compared across groups using log-rank tests. Cox proportional hazards models were used to evaluate the association between clinical or molecular variables and DFS or OS. Proportional hazards assumptions were assessed using Schoenfeld residuals. Variables significantly associated with outcome in univariate analysis (p < 0.05), along with established prognostic factors in localised HNSCC, were included in the multivariable model. These included T and N stage, age at diagnosis, HPV status, and sex. Hazard ratios (HR) with 95% confidence intervals (CI) and p-values were reported. To mitigate the risk of overfitting arising from a low events-per-variable ratio, a robust modeling framework was implemented. We used a Ridge-penalized Cox regression^20^; clinical variables established in the literature (T and N stages, HPV status) were included without penalization to ensure full adjustment. Model stability and predictive performance were assessed using a double-bootstrap procedure, comprising B=1,000 outer iterations for confidence interval estimation and K=500 inner iterations for optimism correction. This approach allowed for the derivation of an optimism-corrected concordance index (C-index) and the construction of robust 95% confidence intervals^21^. All analyses were performed in R (v.4.4.1), using survival, glmnet packages.

**Methods References**

1.	Moreira, A. *et al.* Prognostic value of tumor mutational burden in patients with oral cavity squamous cell carcinoma treated with upfront surgery. *ESMO Open* **6**, 100178 (2021).

2.	Shen, R. & Seshan, V. E. FACETS: allele-specific copy number and clonal heterogeneity analysis tool for high-throughput DNA sequencing. *Nucleic Acids Res* **44**, e131 (2016).

3.	Gao, J. *et al.* Integrative analysis of complex cancer genomics and clinical profiles using the cBioPortal. *Sci Signal* **6**, pl1 (2013).

4.	Chakravarty, D. *et al.* OncoKB: A Precision Oncology Knowledge Base. *JCO Precis Oncol* 1–16 (2017) doi:10.1200/PO.17.00011.

5.	Sanchez-Vega, F. *et al.* Oncogenic Signaling Pathways in The Cancer Genome Atlas. *Cell* **173**, 321-337.e10 (2018).

6.	Tate, J. G. *et al.* COSMIC: the Catalogue Of Somatic Mutations In Cancer. *Nucleic Acids Res* **47**, D941–D947 (2019).

7.	Canisius, S., Martens, J. W. M. & Wessels, L. F. A. A novel independence test for somatic alterations in cancer shows that biology drives mutual exclusivity but chance explains most co-occurrence. *Genome Biol* **17**, 261 (2016).

8.	Marret, G. *et al.* Genomic Alterations in Head and Neck Squamous Cell Carcinoma: Level of Evidence According to ESMO Scale for Clinical Actionability of Molecular Targets (ESCAT). *JCO Precis Oncol* 215–226 (2021) doi:10.1200/PO.20.00280.

9.	Gillis, S. & Roth, A. PyClone-VI: scalable inference of clonal population structures using whole genome data. *BMC Bioinformatics* **21**, 571 (2020).

10.	Nik-Zainal, S. *et al.* The Life History of 21 Breast Cancers. *Cell* **149**, 994–1007 (2012).

11.	Leshchiner, I. *et al.* Comprehensive analysis of tumour initiation, spatial and temporal progression under multiple lines of treatment. 508127 Preprint at https://doi.org/10.1101/508127 (2019).

12.	Abécassis, J., Reyal, F. & Vert, J.-P. CloneSig can jointly infer intra-tumor heterogeneity and mutational signature activity in bulk tumor sequencing data. *Nat Commun* **12**, 5352 (2021).

13.	Dentro, S. C. *et al.* Characterizing genetic intra-tumor heterogeneity across 2,658 human cancer genomes. *Cell* **184**, 2239-2254.e39 (2021).

14.	Dobin, A. *et al.* STAR: ultrafast universal RNA-seq aligner. *Bioinformatics* **29**, 15–21 (2013).

15.	Love, M. I., Huber, W. & Anders, S. Moderated estimation of fold change and dispersion for RNA-seq data with DESeq2. *Genome Biology* **15**, 550 (2014).

16.	Robinson, M. D., McCarthy, D. J. & Smyth, G. K. edgeR: a Bioconductor package for differential expression analysis of digital gene expression data. *Bioinformatics* **26**, 139–140 (2010).

17.	Subramanian, A. *et al.* Gene set enrichment analysis: A knowledge-based approach for interpreting genome-wide expression profiles. *Proceedings of the National Academy of Sciences* **102**, 15545–15550 (2005).

18.	Martínez-Ruiz, C. *et al.* Genomic–transcriptomic evolution in lung cancer and metastasis. *Nature* **616**, 543–552 (2023).

19.	Becht, E. *et al.* Estimating the population abundance of tissue-infiltrating immune and stromal cell populations using gene expression. *Genome Biol* **17**, 1–20 (2016).

20.	Friedman, J., Hastie, T. & Tibshirani, R. Regularization Paths for Generalized Linear Models via Coordinate Descent. *J Stat Softw* **33**, 1–22 (2010).

21.	Noma, H., Shinozaki, T., Iba, K., Teramukai, S. & Furukawa, T. A. Confidence intervals of prediction accuracy measures for multivariable prediction models based on the bootstrap-based optimism correction methods. *Statistics in Medicine* **40**, 5691–5701 (2021).
